# Supplementary material for: Contrasting effects of acute and chronic stress on the transcriptome, epigenome, and immune response of Atlantic salmon
Source: Epigenetics. 2018 Dec 13;13(12):1191–207. doi: 10.1080/15592294.2018.1554520 (PMC6986783; doi:10.1080/15592294.2018.1554520)
Supplement: Supplemental Material [file kepi-13-12-1554520-s001.pdf]

SUPPORTING INFORMATION

Contrasting effects of acute and chronic stress on the transcriptome, epigenome, and on immune response of Atlantic salmon

Tamsyn M. Uren Webster[1], Deiene Rodriguez-Barreto[1], Samuel A.M. Martin[2], Cock van Oosterhout[3], Pablo Orozco-terWengel[4], Joanne Cable[4], Alastair Hamilton[5], Carlos Garcia de Leaniz[1], Sofia Consuegra[1]

1. College of Science, Swansea University, SA2 8PP, UK
2. College of Life Sciences & Medicine, University of Aberdeen, AB24 2TZ, UK
3. School of Environmental Sciences, University of East Anglia, NR4 7TJ, UK
4. School of Biosciences, Cardiff University CF10 3AX, UK
5. Landcatch Natural Selection Ltd, Stirling, FK9 4NF, UK.

This supporting information contains:

Page 2: Supplementary methods
Page 5: Table S1. Morphometric data collected throughout the experiment
Page 6: Table S2. Summary of statistics for RNA-seq libraries
Page 7: Table S3. Differentially expressed genes in acutely stressed fish
Page 8: Table S4. Differentially expressed genes in chronically stressed fish.
Page 12: Table S5. Selected immune genes differentially regulated by LPS
Page 16: Table S6. Summary of statistics for RRBS libraries
Page 17: Table S7. Genes with altered expression and p.promoter methylation in stress groups
Page 19: Table S8. Genes with altered expression and gene body methylation in stress groups
Page 21: Figure S1. Enrichment analysis for DE genes in chronically-stressed fish
Page 22: Figure S2. PCA plot illustrating transcriptomic response to 20 µg/ml LPS.
Page 23: Figure S3. Heat map illustrating transcriptomic response to LPS
Page 24: Figure S4. Enrichment analysis for DE genes in response to LPS exposure
Page 25: Figure S5. Enrichment analysis for genes for which a significant interaction between stress and LPS response was identified
Page 26: Figure S6. Functional enrichment analysis of genes in which DM CpGs were identified
Page 27: Figure S7. Enrichment analysis for genes for which stress altered both expression and DNA methylation
Page 28: Figure S8. Transcription and methylation data of genes for which a significant interaction between stress and transcriptional response to LPS was identified

Supplementary methods

Fish maintenance

Each experimental group (controls, acute stress and chronic stress) consisted of two replicate vertical tray incubators (each containing 500 eggs), and larvae from each replicate tray were transferred to a separate replicate fry trough (length 100 cm, width 40 cm, depth 8 cm) following hatching (at 475 degree days, DD). Egg incubators and fry troughs were supplied with a constant flow (~ 3 L/min) of aerated de-chlorinated tap water in a recirculation system. Fry were offered food prior to the start of feeding (at 850 DD), and fed Nutraplus salmonid feed (Skretting, UK). Water oxygen saturation was maintained above 90%, and ammonia (<0.02 mg/L), nitrite (<0.01 mg/L), nitrate (<15 mg/L) and pH (7.5 ± 0.2) levels were maintained within the optimal range for the species. Water temperature was increased from 9 °C to 11 °C (0.5 °C increase per month) and photoperiod was adjusted from 10:14h to 14:10h light: dark over the 4 month experimental period, to reflect seasonal variation. At each sampling point fish were euthanised via an overdose of anaesthetic (Phenoxyethanol; 0.5 mg/L), followed by destruction of the brain according to UK Home Office regulations.

Transcriptome and methylome sequencing

RNA and DNA were simultaneously extracted using the Qiagen AllPrep DNA/RNA Mini Kit, according to the manufacturer's instructions and then nucleic acid concentration was assessed using a Qubit 3.0 Fluorometer (Thermo Fisher Scientific, UK).

Transcriptomic analysis was conducted using RNA-seq. RNA purity and integrity was assessed using a NanoDrop NS-100 Spectrophotometer (NanoDrop Technologies, USA) and an Agilent 2100 Bioanalyzer (Agilent Technologies, USA). All RNA used for library construction was of high quality with 260/230 and 260/280 ratios > 1.8 and RIN scores >8. Library preparation was performed using the Illumina TruSeq RNA Library Prep Kit v2, with 1 µg total input RNA. Sequencing of the 48 libraries was performed on an Illumina NextSeq500 platform at University of Aberdeen, across a total of eight flow cells, using 76 bp paired-end sequencing.

Methylation analysis was performed using Reduced Representation Bisulfide Sequencing (RRBS). Bisulphite converted genomic DNA libraries were prepared using the Diagenode Premium RRBS Kit, including fully methylated and un-methylated spike-in controls to monitor bisulphite conversion efficiencies. Briefly, 100 ng of genomic DNA were digested with *MspI*, followed by end-repair, adaptor ligation and bead-based fragment size selection. 24 samples were multiplexed in pools of six following the manufacturers' recommendations. After multiplexing, the pooled samples were bisulphite-converted, amplified by enrichment PCR, and quality checked using the Agilent D1000 ScreenTape System. The libraries were then sequenced across 6 lanes on Illumina NextSeq 500 platform at Cardiff University using 76 bp single-end sequencing.

Transcriptomic analysis

Quality screening of raw reads was performed using FastQC (Andrews 2010). Trimmomatic (Bolger et al. 2014) was used to remove contaminating adaptor sequences and the last three bp of all reads (containing nucleotide bias), and trim poor quality bases from the 3' end using a sliding window of 4 bp, and specifying Q≥20. All reads < 30 bp in length were discarded. High quality paired reads from each sample were then aligned to the Atlantic salmon genome (v GCF_000233375.1_ICSAG_v2; (Davidson et al. 2010; Lien et al. 2016)) using HISAT2 (v 2.1.0; (Kim et al. 2015)), after first converting the GFF3 annotation file to a GTF file using gffread (Pertea 2016), to enable splice-aware alignment and novel transcript discovery. SAM alignment files were converted to sorted BAM files using samtools v 1.5 (Li et al. 2009), and transcript reconstruction and assembly was then performed using StringTie (v1.3.3) using recommended parameters (Pertea et

al. 2015). Non-normalised read counts for the set of merged assembled transcripts were then extracted using the prepDE.py script supplied in the StringTie package.

Differential expression analysis was performed using DeSeq2 (Love et al. 2014) for 78,229 putative loci with at least 1 read count in more than one sample. Differentially expressed genes in response to stress and LPS exposure were identified using a multifactorial design, including the main effects of stress and LPS exposure, and their interaction, and accounting for potential variation between replicate tanks. Within the model, independent filtering of loci with low coverage depth was applied, optimising power for identification of differentially expressed transcripts at a threshold of alpha=0.05. Default settings were applied for outlier detection and moderation of gene-wise dispersion estimates. Genes were considered significantly differentially expressed at FDR <0.05, and the contrast function was used to extract differentially expressed gene identities.

For visualisation and cluster analysis, raw counts were normalised using variance stabilising transformation within DeSeq2. Multidimensional scaling (MDS) analysis was performed using pairwise distances between all samples, and visualised using ggplot2 (Wickham 2009). Heat maps were constructed to visualise transcriptional response to LPS, and the effect of each type of stress on transcriptional response to LPS. Hierarchical clustering of all genes significantly regulated by LPS, and all genes for which a significant interaction between stress and LPS response was identified, was performed using an Euclidean distance metric and visualised using the Pheatmap package in R (Kolde 2015). Functional enrichment analysis of differentially regulated genes was performed using DAVID (v 6.8; (Huang et al. 2008)), using all expressed genes in the gill transcriptome as background, and considering terms significantly enriched with q<0.05 after multiple test correction (Benjamini-Hochberg). Due to the lack of gene ontology terms attached to Atlantic salmon gene ids, the enrichment analysis was conducted on zebrafish orthologs which were identified using Blastx against the Uniprot peptide database (specifying a minimum e value cutoff of $1e^{-15}$).

Methylation analysis

Following initial quality assessment of raw sequences using FastQC (Andrews 2010), TrimGalore (Kreger 2016) was used to trim low-quality base calls and adapter contamination using the '-- rrbs' option and specifying Q≥20. High quality reads were aligned to the Atlantic salmon reference genome and cytosine methylation calls extracted using Bismark v 0.17.0 (Krueger & Andrews 2011) using the '--comprehensive' parameter. Mapped data were then processed using SeqMonk (Andrews 2007) within R, considering only methylation within CpG context. Only CpGs with a minimum coverage of 10 reads in each of the 24 samples were included in the analysis, and extreme coverage outliers (>99.9th percentile) were removed. CpG island locations were predicted using the newcpgreport tool within EMBOSS (Rice et al. 2000), defining islands as regions of ≥200 bp with GC % ≥ 55% and an observed-to-expected CpG ratio of ≥ 65%, island shores as regions located up to 2 kb of a CpG island, and shelves as 2 kb regions from a CpG shore.

Differentially methylated CpGs (DMCpGs) were identified using logistic regression tests on the quantitated normalised data based on FDR<0.01 after multiple testing correction (Benjamini-Hochberg) and >20% minimal CpG methylation difference (|ΔM|). For each DMCpG, we identified the genomic location (within gene body, promoter region (≤1500 bp upstream of the transcription start site (TSS)), or intergenic region) and the context location (CpG island, CpG shore, CpG shelf). For the DMCpGs that were within a gene, or within 2 kb (upstream or downstream) of the TSS or transcription termination site (TTS) respectively, we also performed enrichment analysis based on gene function using DAVID v 6.8 (Huang et al. 2008), after converting to zebrafish ids as described above. To generate a more stringent list of DMCpGs for further cluster analysis between stress groups, we additionally ran t-tests for each paired comparison using a threshold of p<0.01, to identify DMCpGs shared by both statistical methods.

References

Andrews, S. 2007 SeqMonk: A tool to visualise and analyse high throughput mapped sequence data. https://www.bioinformatics.babraham.ac.uk/projects/seqmonk/.

Andrews, S. 2010 FastQC A Quality Control tool for High Throughput Sequence Data. http://www.bioinformatics.babraham.ac.uk/projects/fastqc/.

Bolger, A. M., Lohse, M. & Usadel, B. 2014 Trimmomatic: a flexible trimmer for Illumina sequence data. *Bioinformatics* **30**, 2114-20.

Davidson, W. S., Koop, B. F., Jones, S. J., Iturra, P., Vidal, R., Maass, A., Jonassen, I., Lien, S. & Omholt, S. W. 2010 Sequencing the genome of the Atlantic salmon *(Salmo salar*). *Genome Biology* **11**, 403-410.

Huang, D. W., Sherman, B. T. & Lempicki, R. A. 2008 Systematic and integrative analysis of large gene lists using DAVID bioinformatics resources. *Nature Protocols* **4**, 44-57.

Kim, D., Langmead, B. & Salzberg, S. L. 2015 HISAT: a fast spliced aligner with low memory requirements. *Nature Methods* **12**, 357-360.

Kolde, R. 2015 Pretty Heatmaps. *R package version 3.1-131*.

Kreger, F. 2016 TrimGalore. A wrapper around Cutadapt and FastQC to consistently apply adapter and quality trimming to FastQ files, with extra functionality for RRBS data. https://www.bioinformatics.babraham.ac.uk/projects/trim_galore/.

Krueger, F. & Andrews, S. R. 2011 Bismark: a flexible aligner and methylation caller for Bisulfite-Seq applications. *Bioinformatics* **27**, 1571-1572.

Li, H., Handsaker, B., Wysoker, A., Fennell, T., Ruan, J. & Homer, N. 2009 The Sequence Alignment/Map format and SAMtools. *Bioinformatics* **25**, 2078-2079.

Lien, S., Koop, B. F., Sandve, S. R., Miller, J. R., Kent, M. P., Nome, T., Hvidsten, T. R., Leong, J. S., Minkley, D. R., Zimin, A., Grammes, F., Grove, H., Gjuvsland, A., Walenz, B., Hermansen, R. A., von Schalburg, K., Rondeau, E. B., Di Genova, A., Samy, J. K. A., Olav Vik, J., Vigeland, M. D., Caler, L., Grimholt, U., Jentoft, S., Inge Våge, D., de Jong, P., Moen, T., Baranski, M., Palti, Y., Smith, D. R., Yorke, J. A., Nederbragt, A. J., Tooming-Klunderud, A., Jakobsen, K. S., Jiang, X., Fan, D., Hu, Y., Liberles, D. A., Vidal, R., Iturra, P., Jones, S. J. M., Jonassen, I., Maass, A., Omholt, S. W. & Davidson, W. S. 2016 The Atlantic salmon genome provides insights into rediploidization. *Nature* **533**, 200-205.

Love, M. I., Huber, W. & Anders, S. 2014 Moderated estimation of fold change and dispersion for RNA-seq data with DESeq2. *Genome Biology* **15**, 550-555.

Pertea, G. 2016 gffread. *avaialble at:* https://github.com/gpertea/gffread.

Pertea, M., Pertea, G. M., Antonescu, C. M., Chang, T.-C., Mendell, J. T. & Salzberg, S. L. 2015 StringTie enables improved reconstruction of a transcriptome from RNA-seq reads. *Nature Biotechnology* **33**, 290-295.

Rice, P., Longden, I. & Bleasby, A. 2000 EMBOSS: the European Molecular Biology Open Software Suite. *Trends in Genetics* **16**, 276-277.

Wickham, H. 2009 *ggplot2: Elegant Graphics for Data Analysis*: Springer-Verlag New York.

Table S1. Morphometric data collected throughout the experiment.

	Control A	Control B	Chronic stress A	Chronic stress B	Acute stress A	Acute stress B	Statistical significance
Hatching rate (%)	94.6	96.6	96.6	95.2	94.0	94.6	$F_{3,2}$=1.38, P=0.377
Post-hatch mortality rate (%)	11.8	10.8	10.2	11.8	9.2	11.8	$F_{3,2}$=0.19, P=0.836
Mass (mg) 492 DD	187.4 ±6.2	185.5 ±10.0	183.3 ±3.5	185.5 ±7.8	183.3 ±6.2	189.6 ±4.6	$F_{3,114}$=0.37, P=0.718
Mass (mg) 748 DD	194.3 ±15.2	189.5 ±15.5	156.9 ±13.8	168.6 ±16.6	197.9 ±27.3	189.6 ±19.0	$F_{3,114}$=15.82, P=0.025*
Mass (mg) 1019 DD	236.2 ±24.6	246.3 ±37.0	216.7 ±28.3	200.6 ±34.7	249.6 ±34.5	256.6 ±34.8	$F_{3,114}$=15.42, P=0.026*
Mass (mg) 1323 DD	287.9 ±43.1	337.0 ±29.8	281.5 ±26.2	262.2 ±27.8	310.7 ±62.6	290.5 ±56.6	$F_{3,114}$=1.63, P=0.330
Mass (mg) 1532 DD	381.0 ±73.1	329.8 ±102	348.9 ±76.3	329.9 ±72.5	376.1 ±110	410.7 ±96.1	$F_{3,114}$=4.78, P=0.117
Length (cm) 1532 DD	3.54 ±0.19	3.58 ±0.28	3.50 ±0.31	3.39 ±0.26	3.47 ±0.26	3.62 ±0.28	$F_{3,114}$=1.36, P=0.381
Condition factor (1532 DD)	0.85 ±0.09	0.84 ±0.09	0.81 ±0.11	0.83 ±0.07	0.86 ±0.07	0.85 ±0.05	$F_{3,114}$=2.18, P=0.260

Hatching rate and mortality rate; n=2 replicate tanks/ treatment. Mass and condition factor; n=20 individuals per tank, values presented are mean ±SD. Asterisks indicate significant stress effect (P< 0.05, using linear mixed effect models).

Table S2. Summary of statistics for RNA-seq libraries

Group	Sample ID	Paired raw reads (millions)	QC reads (millions)	Total alignment (%)	Unique alignment %	Library size factor
Control	48	32.62	30.01	94.48	83.14	1.03
Control	16	32.79	30.27	95.72	85.54	1.05
Control	47	34.11	31.25	94.70	84.16	1.07
Control	45	34.99	31.85	93.64	83.89	1.10
Control	15	36.10	33.74	93.79	83.02	1.15
Control	17	36.94	34.16	94.35	83.64	1.17
Control	18	39.01	35.99	94.11	83.47	1.23
Control	46	39.57	36.51	95.64	85.10	1.27
Substrate stress	36	29.15	26.46	94.85	84.34	0.91
Substrate stress	39	31.91	28.93	95.91	85.15	1.02
Substrate stress	40	32.10	29.97	95.41	84.43	1.05
Substrate stress	10	34.42	31.24	96.26	86.13	1.10
Substrate stress	37	36.94	34.13	94.57	83.91	1.17
Substrate stress	9	38.58	34.95	95.40	84.97	1.21
Substrate stress	35	38.79	35.93	94.87	82.49	1.25
Substrate stress	38	40.49	37.09	95.22	83.71	1.30
Cold shock	57	34.89	31.48	94.98	84.40	1.09
Cold shock	26	38.48	34.02	93.18	83.15	1.14
Cold shock	58	37.88	34.80	93.24	82.61	1.17
Cold shock	28	41.83	37.81	94.75	83.93	1.31
Cold shock	27	41.60	37.98	93.35	83.04	1.29
Cold shock	55	43.99	39.19	94.67	83.86	1.35
Cold shock	56	43.70	39.96	93.57	82.89	1.35
Cold shock	25	44.60	40.79	93.35	83.01	1.38
LPS Control	113	16.13	14.56	85.4	75.99	0.47
LPS Control	115	14.32	13.07	93.8	83.52	0.43
LPS Control	117	21.81	20.61	93.64	82.88	0.71
LPS Control	118	22.74	20.96	94.56	84.01	0.72
LPS Control	149	19.50	17.98	95.63	84.36	0.60
LPS Control	150	14.93	13.80	96.01	85.51	0.45
LPS Control	152	29.54	27.28	96.76	85.87	0.91
LPS Control	153	37.18	34.42	86.06	75.81	1.08
LPS Substrate stress	103	29.94	27.00	96.36	86.23	0.86
LPS Substrate stress	104	17.64	16.12	97.03	86.11	0.53
LPS Substrate stress	105	29.32	26.83	95.33	85.61	0.92
LPS Substrate stress	106	32.50	29.87	97.06	86.56	1.03
LPS Substrate stress	137	27.58	25.65	96.71	86.56	0.87
LPS Substrate stress	138	40.05	37.69	96.66	85.77	1.27
LPS Substrate stress	139	28.82	26.60	96.73	85.93	0.89
LPS Substrate stress	142	25.63	23.56	89.23	78.89	0.77
LPS Cold shock	126	30.41	27.50	95.51	85.79	0.89
LPS Cold shock	127	31.24	28.75	93.33	82.93	0.91
LPS Cold shock	128	35.76	32.70	94.95	85.12	1.07
LPS Cold shock	130	30.61	28.16	92.93	82.41	0.96
LPS Cold shock	161	25.79	23.54	94.18	83.04	0.74
LPS Cold shock	163	35.55	32.45	97.07	85.67	1.11
LPS Cold shock	165	32.67	30.98	84.98	75.26	0.97
LPS Cold shock	166	23.26	21.57	85.10	75.74	0.67
Mean		*30.23*	*27.83*	*94.54*	*84.13*	

Table S3. Differentially expressed genes in fish subject to acute stress compared to the control group (FDR <0.05). Shading indicates up (red) or down (green) regulation.

Accession	Log2 Fold Change	FDR	Gene name	Gene description
XR_001329667.1	-5.59	3.76E-06	LOC106609253	uncharacterized ncRNA
XM_014133628.1	2.82	3.85E-02	LOC106565937	tumor necrosis factor receptor superfamily member 21-like 2C transcript variant X1
XLOC_010727	2.62	3.87E-02	XLOC_010727	na
XLOC_070250	-3.84	3.88E-02	XLOC_070250	na
XLOC_085183	3.65	3.89E-02	XLOC_085183	na
XM_014198068.1	-3.26	3.91E-02	LOC106603863	syntaxin-11-like
XLOC_005326	-5.30	3.93E-02	XLOC_005326	na
XLOC_067368	3.87	4.02E-02	XLOC_067368	na
XLOC_022690	5.77	4.07E-02	XLOC_022690	na
XM_014206976.1	-0.77	4.09E-02	ufsp1	UFM1-specific peptidase 1 (non-functional)
XLOC_016360	-7.81	4.09E-02	XLOC_016360	na
XM_014139260.1	1.40	4.11E-02	LOC106568695	probable G-protein coupled receptor 158
XLOC_083835	11.93	4.15E-02	XLOC_083835	na
XM_014205335.1	-2.70	4.17E-02	LOC106607875	tumor necrosis factor receptor superfamily member 21-like
XM_014129398.1	1.68	4.26E-02	LOC106563640	putative defense protein Hdd11
XM_014195313.1	-2.73	4.47E-02	LOC106602593	sodium/potassium/calcium exchanger 2-like2C transcript variant X1
XLOC_055448	2.47	4.48E-02	XLOC_055448	na
XM_014177758.1	1.99	4.78E-02	LOC106588601	mucin-5AC-like
XLOC_063470	1.34	4.98E-02	XLOC_063470	na

Table S4. Differentially expressed genes in fish subject to chronic stress compared to the control group (FDR <0.05). Shading indicates up (red) or down (green) regulation.

Accession	Log2 Fold Change	FDR	Gene name	Gene description
XLOC_057717	-29.17	0.00E+00	XLOC_057717	na
XM_014124956.1	5.54	3.89E-08	LOC106561235	gastricsin-like 2C transcript variant X1
XM_014147385.1	4.90	3.89E-08	LOC106572851	pepsin A-like
XM_014124958.1	6.12	5.58E-07	LOC106561236	gastricsin-like
XLOC_042823	2.93	7.18E-07	XLOC_042823	na
XLOC_049505	4.18	1.91E-06	XLOC_049505	na
XR_001329667.1	-5.58	6.59E-06	LOC106609253	uncharacterized LOC106609253
XM_014166867.1	5.47	7.19E-06	LOC106583076	pepsin A-like 2C transcript variant X1
XM_014208287.1	3.50	1.04E-05	LOC106609441	hemicentin-1-like
XLOC_000030	1.49	3.39E-03	XLOC_000030	na
NM_001279140.1	1.99	5.02E-03	igfbp-1a1	insulin-like growth factor binding protein 1 paralog A1
XM_014131771.1	6.79	5.02E-03	LOC106565088	acidic mammalian chitinase-like
XM_014132502.1	0.93	5.02E-03	LOC106565406	nuclear receptor subfamily 4 group A member 1-like 2C transcript variant X1
XM_014146514.1	1.01	5.02E-03	LOC106572395	F-box only protein 6-like 2C transcript variant X1
XM_014149916.1	2.22	5.02E-03	LOC106574225	four and a half LIM domains protein 2-like 2C transcript variant X1
XM_014160844.1	1.25	5.02E-03	LOC106580132	uncharacterized LOC106580132
XM_014162186.1	1.30	5.02E-03	LOC106580780	smoothelin-like protein 2
XM_014171431.1	1.42	5.02E-03	LOC106585340	immediate early response gene 5-like protein
XR_001328272.1	3.72	5.02E-03	LOC106602674	uncharacterized LOC106602674
XM_014201340.1	4.49	5.02E-03	LOC106605555	cornifelin homolog
NM_001141877.1	1.31	5.02E-03	sc61g	transport protein Sec61 subunit gamma
XLOC_001214	1.63	5.02E-03	XLOC_001214	na
XLOC_005379	1.81	5.02E-03	XLOC_005379	na
XLOC_033969	1.54	5.02E-03	XLOC_033969	na
XM_014200905.1	1.84	5.96E-03	LOC106605369	uncharacterized LOC106605369 2C transcript variant X1
XM_014140363.1	1.39	6.15E-03	LOC106569220	protein transport protein Sec61 subunit gamma-like 2C transcript variant X1
XR_001320676.1	1.69	6.21E-03	LOC106570462	uncharacterized LOC106570462
XLOC_009411	1.25	1.31E-02	XLOC_009411	na
XM_014195962.1	1.50	2.13E-02	LOC106602938	NADH dehydrogenase [ubiquinone] 1 alpha subcomplex subunit 2-like
NM_001141530.2	-1.19	2.38E-02	itl1a	Intelectin-1a
XLOC_093138	1.25	2.49E-02	XLOC_093138	na
XR_001319213.1	1.76	2.88E-02	LOC106563242	uncharacterized LOC106563242
XM_014212854.1	0.89	3.02E-02	hprt1	hypoxanthine phosphoribosyltransferase 1 2C transcript variant X1
XM_014173392.1	1.56	3.02E-02	LOC100137092	dopachrome tautomerase-like
XM_014126463.1	-2.74	3.02E-02	LOC106561968	cathepsin L1-like
XM_014127049.1	1.89	3.02E-02	LOC106562278	dysbindin domain-containing protein 1-like 2C transcript variant X1
XM_014139157.1	-0.84	3.02E-02	LOC106568655	erythroferrone-like
XM_014141306.1	1.64	3.02E-02	LOC106569732	collagen alpha-1(VI) chain-like
XM_014178515.1	0.94	3.02E-02	LOC106588942	tissue factor pathway inhibitor 2-like
XM_014183729.1	-0.72	3.02E-02	LOC106592390	E3 ubiquitin-protein ligase TRIM39-like
XM_014193844.1	-0.54	3.02E-02	LOC106601548	uncharacterized LOC106601548 2C transcript variant X1
NM_001123607.1	1.88	3.02E-02	mrf4	myogenic regulatory factor 4
XLOC_000028	1.59	3.02E-02	XLOC_000028	na
XLOC_041258	1.60	3.02E-02	XLOC_041258	na
XM_014204439.1	1.39	3.10E-02	LOC106607463	interferon a3-like 2C transcript variant X1
XLOC_073991	-1.04	3.10E-02	XLOC_073991	na
NM_001139619.1	1.09	3.47E-02	eif4e1a	eukaryotic translation initiation factor 4e 1a
NM_001140868.1	1.37	3.56E-02	cf125	CF125 protein
NM_001141156.1	0.86	3.56E-02	hint1	histidine triad nucleotide binding protein 1
XM_014124782.1	1.32	3.56E-02	hrsp12	heat-responsive protein 12
XM_014126616.1	1.18	3.56E-02	LOC106562066	trafficking protein particle complex subunit 2-like protein

XR_001319149.1	1.14	3.56E-02	LOC106562957	uncharacterized LOC106562957 2C transcript variant X1
XM_014131619.1	1.34	3.56E-02	LOC106565035	cytochrome c oxidase subunit 6C-1 2C transcript variant X1
XM_014131770.1	6.84	3.56E-02	LOC106565087	acidic mammalian chitinase-like
XM_014135804.1	0.80	3.56E-02	LOC106567025	translocon-associated protein subunit delta-like
XM_014137127.1	1.07	3.56E-02	LOC106567624	exocyst complex component 3-like
XM_014138035.1	1.23	3.56E-02	LOC106568029	60S ribosomal protein L36a-like
XM_014143322.1	1.36	3.56E-02	LOC106570789	DNA-directed RNA polymerases I 2C II 2C and III subunit RPABC4-like 2C
XM_014144577.1	1.13	3.56E-02	LOC106571451	signal recognition particle 9 kDa protein-like
XR_001320980.1	1.94	3.56E-02	LOC106571698	uncharacterized LOC106571698
XM_014156332.1	1.54	3.56E-02	LOC106577897	plasminogen activator inhibitor 1-like
XR_001322807.1	1.34	3.56E-02	LOC106580131	uncharacterized LOC106580131
XM_014162429.1	0.89	3.56E-02	LOC106580897	uncharacterized LOC106580897
XM_014169453.1	1.23	3.56E-02	LOC106584322	torsin-3A-like 2C transcript variant X1
XM_014176091.1	1.04	3.56E-02	LOC106587574	nuclear envelope phosphatase-regulatory subunit 1 2C transcript variant X1
XM_014176111.1	1.33	3.56E-02	LOC106587582	SS18-like protein 2
XM_014177694.1	0.71	3.56E-02	LOC106588561	DNA-directed RNA polymerase III subunit RPC7-like
XM_014183834.1	1.94	3.56E-02	LOC106592479	DNA damage-inducible transcript 4 protein-like
XM_014185875.1	1.22	3.56E-02	LOC106594505	troponin I 2C fast skeletal muscle-like
XM_014186156.1	1.04	3.56E-02	LOC106594764	zinc finger protein 658B-like
XM_014192782.1	-1.07	3.56E-02	LOC106600973	beta-1 2C4-mannosyl-glycoprotein 4-beta-N-acetylglucosaminyltransferase-like
XM_014193023.1	-0.54	3.56E-02	LOC106601099	myoferlin-like
XM_014194484.1	1.53	3.56E-02	LOC106602033	cofilin-2-like
XM_014196358.1	1.04	3.56E-02	LOC106603114	LRRN4 C-terminal-like protein 2C transcript variant X1
XM_014204008.1	1.71	3.56E-02	LOC106607240	transmembrane protein 98-like 2C transcript variant X1
XM_014210981.1	1.57	3.56E-02	LOC106611115	intron-binding protein aquarius-like
XM_014211770.1	1.26	3.56E-02	LOC106611504	60S ribosomal protein L36a-like
XM_014212334.1	1.00	3.56E-02	LOC106611775	short coiled-coil protein B-like 2C transcript variant X1
NM_001140861.2	1.39	3.56E-02	scam4	Secretory carrier-associated membrane protein 4
XLOC_000027	1.56	3.56E-02	XLOC_000027	na
XLOC_042919	1.99	3.56E-02	XLOC_042919	na
XLOC_075460	1.04	3.56E-02	XLOC_075460	na
NM_001141187.1	0.94	3.56E-02	ykt6	YKT6 v-SNARE homolog (S. cerevisiae)
XM_014191134.1	0.80	3.68E-02	ube2v2	ubiquitin-conjugating enzyme E2 variant 2 2C transcript variant X1
XR_001322400.1	1.21	3.86E-02	LOC106578263	uncharacterized LOC106578263
XM_014181663.1	1.32	3.86E-02	LOC106590555	protein transport protein Sec61 subunit gamma 2C transcript variant X1
XR_001325958.1	1.34	3.86E-02	LOC106593774	uncharacterized LOC106593774
XLOC_064668	1.01	3.86E-02	XLOC_064668	na
XLOC_035802	1.26	3.90E-02	XLOC_035802	na
XM_014192152.1	0.82	4.03E-02	fam173a	family with sequence similarity 173 2C member A 2C transcript variant X1
XM_014127899.1	1.96	4.03E-02	gatm	glycine amidinotransferase (L-arginine:glycine amidinotransferase)
XM_014147886.1	0.80	4.03E-02	LOC106573170	protein C19orf12 homolog 2C transcript variant X1
XM_014155116.1	1.88	4.03E-02	LOC106577211	DNA damage-inducible transcript 4 protein-like
XM_014157473.1	2.34	4.03E-02	LOC106578540	cAMP-responsive element modulator-like 2C transcript variant X1
XR_001325901.1	1.33	4.03E-02	LOC106593518	uncharacterized LOC106593518
NM_001141193.2	0.87	4.03E-02	pigy	phosphatidylinositol glycan anchor biosynthesis 2C class Y
NM_001139699.1	1.57	4.03E-02	ppdpf	c20orf149 protein
XM_014206938.1	1.32	4.03E-02	tyrp-1b	tyrosinase-related protein 1b
NM_001279145.1	1.27	4.16E-02	igfbp-6a2	insulin-like growth factor binding protein 6 paralog A2
NM_001139734.1	1.13	4.16E-02	LOC100194650	ribosomal protein L36a-2
XM_014124215.1	1.47	4.16E-02	LOC106560846	leucine-rich repeat-containing protein 20-like
XR_001319428.1	-3.04	4.16E-02	LOC106564200	uncharacterized LOC106564200
XM_014137047.1	0.73	4.16E-02	LOC106567574	ubiquitin carboxyl-terminal hydrolase 2-like 2C transcript variant X1
XM_014138326.1	1.21	4.16E-02	LOC106568198	cytochrome b-c1 complex subunit 9-like
XM_014141876.1	1.19	4.16E-02	LOC106570015	NADH dehydrogenase [ubiquinone] iron-sulfur protein 5-like
XM_014142851.1	1.29	4.16E-02	LOC106570471	26S proteasome complex subunit DSS1-like
XM_014148796.1	0.79	4.16E-02	LOC106573605	serine/threonine-protein kinase STK11-like 2C transcript variant X1

XM_014150737.1	1.34	4.16E-02	LOC106574722	small integral membrane protein 11-like
XM_014152748.1	1.14	4.16E-02	LOC106575974	protein S100-A1-like 2C transcript variant X1
XM_014156134.1	0.92	4.16E-02	LOC106577792	39S ribosomal protein L52 2C mitochondrial-like 2C transcript variant X1
XM_014160435.1	1.02	4.16E-02	LOC106579969	prostaglandin E synthase 2-like 2C transcript variant X1
XM_014166227.1	0.98	4.16E-02	LOC106582796	tumor suppressor candidate 2-like 2C transcript variant X1
XM_014167009.1	0.91	4.16E-02	LOC106583154	musculoskeletal embryonic nuclear protein 1-like
XR_001324158.1	1.18	4.16E-02	LOC106586240	uncharacterized LOC106586240
XM_014175164.1	1.05	4.16E-02	LOC106587124	CD59 glycoprotein-like 2C transcript variant X1
XM_014176249.1	1.08	4.16E-02	LOC106587662	probable G-protein coupled receptor 158 2C transcript variant X1
XM_014176405.1	1.17	4.16E-02	LOC106587763	solute carrier family 22 member 18-like
XM_014178257.1	1.09	4.16E-02	LOC106588813	fatty acid-binding protein 2C heart-like
XM_014178836.1	1.35	4.16E-02	LOC106589133	60S ribosomal protein L30 2C transcript variant X1
XR_001326794.1	1.17	4.16E-02	LOC106596919	uncharacterized LOC106596919
XR_001328811.1	1.41	4.16E-02	LOC106605220	uncharacterized LOC106605220
XR_001329073.1	1.35	4.16E-02	LOC106606515	uncharacterized LOC106606515
XR_001329867.1	1.50	4.16E-02	LOC106610188	uncharacterized LOC106610188
XR_001329944.1	1.00	4.16E-02	LOC106610644	uncharacterized LOC106610644
XM_014211819.1	1.95	4.16E-02	LOC106611527	plasticin-like
XM_014212895.1	0.85	4.16E-02	LOC106612073	tRNA 2'-phosphotransferase 1-like
XM_014137968.1	0.91	4.16E-02	mrpl22	mitochondrial ribosomal protein L22 2C transcript variant X1
XM_014203712.1	1.27	4.16E-02	ndufa6	NADH dehydrogenase (ubiquinone) 1 alpha subcomplex 2C 6 2C 14kDa
XM_014125583.1	-0.38	4.16E-02	papd5	PAP associated domain containing 5 2C transcript variant X1
NM_001141852.1	1.41	4.16E-02	pcbd2	pterin-4 alpha-carbinolamine dehydratase cofactor of hepatocyte factor 1
XM_014167247.1	1.37	4.16E-02	pmel	premelanosome protein
XM_014178471.1	1.07	4.16E-02	shfm1	split hand/foot malformation (ectrodactyly) type 1
XM_014204948.1	1.18	4.16E-02	smim8	small integral membrane protein 8
XLOC_000029	1.44	4.16E-02	XLOC_000029	na
XLOC_019764	1.02	4.16E-02	XLOC_019764	na
XLOC_060760	1.19	4.16E-02	XLOC_060760	na
NM_001140614.1	1.15	4.16E-02	znf593	zinc finger protein 593
NM_001146400.1	1.15	4.26E-02	acot13	acyl-CoA thioesterase 13
NM_001141403.1	1.63	4.26E-02	bag2	BCL2-associated athanogene 2
NM_001140872.1	1.43	4.26E-02	cmc4	C-x(9)-C motif containing 4
NM_001252357.1	1.11	4.26E-02	dad1	defender against cell death 1
XM_014142573.1	0.83	4.26E-02	fpgs	folylpolyglutamate synthase 2C transcript variant X1
NM_001141352.1	0.59	4.26E-02	hlx	H2.0-like homeobox protein
NM_001141159.2	1.09	4.26E-02	leg	Beta-galactoside-binding lectin
XR_001319276.1	1.51	4.26E-02	LOC106563557	uncharacterized LOC106563557
XR_001319318.1	1.08	4.26E-02	LOC106563787	uncharacterized LOC106563787
XM_014130190.1	0.60	4.26E-02	LOC106564164	zinc finger protein 501-like
XM_014140307.1	0.91	4.26E-02	LOC106569195	U6 snRNA-associated Sm-like protein LSm1 2C transcript variant X1
XM_014143450.1	2.09	4.26E-02	LOC106570884	perforin-1-like
XM_014148711.1	1.11	4.26E-02	LOC106573564	mitochondrial import inner membrane translocase subunit Tim13
XM_014152166.1	1.40	4.26E-02	LOC106575576	small integral membrane protein 11-like
XM_014153767.1	0.82	4.26E-02	LOC106576547	dnaJ homolog subfamily C member 2-like
XR_001322942.1	2.38	4.26E-02	LOC106580674	uncharacterized LOC106580674 2C transcript variant X1
XM_014184745.1	-1.98	4.26E-02	LOC106593408	myosin heavy chain 2C fast skeletal muscle-like
XM_014189389.1	2.40	4.26E-02	LOC106598344	apolipoprotein D-like 2C transcript variant X1
XM_014190321.1	1.18	4.26E-02	LOC106599203	tissue factor-like 2C transcript variant X1
XM_014191583.1	0.97	4.26E-02	LOC106600265	NEDD8-like
XM_014192600.1	1.61	4.26E-02	LOC106600870	myosin light chain 4-like
XR_001328026.1	1.15	4.26E-02	LOC106601549	uncharacterized LOC106601549
XM_014203443.1	0.97	4.26E-02	LOC106606948	transmembrane protein 100-like
XM_014136451.1	1.19	4.26E-02	tnfrsf6b	tumor necrosis factor receptor superfamily 2C member 6b 2C decoy 2C transcript variant X1
XLOC_044742	0.99	4.26E-02	XLOC_044742	na

XLOC_050577	0.78	4.26E-02	XLOC_050577	na
XLOC_051847	1.42	4.26E-02	XLOC_051847	na
XLOC_055978	1.02	4.26E-02	XLOC_055978	na
NM_001140335.1	0.86	4.27E-02	chac1	ChaC 2C cation transport regulator-like 1
XM_014159173.1	1.73	4.27E-02	LOC106579339	putative protein PTGES3L
XM_014166913.1	1.00	4.27E-02	LOC106583097	MICOS complex subunit Mic10-like
XM_014202763.1	1.90	4.27E-02	LOC106606507	transmembrane protein 100-like
XM_014207454.1	1.04	4.27E-02	LOC106609071	oligosaccharyltransferase complex subunit ostc 2C transcript variant X1
XM_014212538.1	1.06	4.27E-02	LOC106611894	zinc finger protein 782-like
NM_001141804.1	0.67	4.27E-02	prosc	proline synthetase co-transcribed homolog (bacterial)
NM_001140810.1	1.14	4.29E-02	pfdn5	prefoldin subunit 5
NM_001123601.1	1.32	4.35E-02	myod1c	myoblast determination protein 1c
XR_001318997.1	1.19	4.38E-02	LOC106562222	uncharacterized LOC106562222
XM_014215517.1	0.84	4.38E-02	LOC106613363	intraflagellar transport protein 20 homolog
XM_014165878.1	0.52	4.44E-02	LOC106582633	bladder cancer-associated protein
NM_001165346.1	0.68	4.54E-02	egln2	Egl nine homolog 2
XM_014152959.1	1.06	4.54E-02	LOC106576080	cytochrome c oxidase assembly factor 6 homolog 2C transcript variant X1
XM_014180972.1	1.26	4.55E-02	cssa29h8orf59	chromosome ssa29 open reading frame 2C human C8orf59
XM_014126951.1	1.11	4.55E-02	LOC106562224	probable ribosome biogenesis protein RLP24
XM_014183303.1	1.00	4.55E-02	LOC106592004	UPF0390 protein zgc136864-like
XR_001328547.1	1.00	4.55E-02	LOC106603962	uncharacterized LOC106603962 2C transcript variant X1
XM_014198652.1	1.02	4.55E-02	LOC106604209	ATP synthase subunit O 2C mitochondrial-like 2C transcript variant X1
XLOC_039816	0.56	4.55E-02	XLOC_039816	na
XM_014177637.1	-6.41	4.57E-02	LOC106588522	mucin-2-like
XM_014172709.1	1.66	4.59E-02	hspb3	heat shock 27kDa protein 3
XM_014166242.1	0.85	4.59E-02	LOC106582802	T-cell leukemia translocation-altered gene protein homolog 2C variant X1
XM_014169343.1	0.64	4.61E-02	LOC106584278	peroxiredoxin-6-like 2C transcript variant X1
XR_001320979.1	1.12	4.61E-02	LOC106571693	uncharacterized LOC106571693
XM_014154124.1	0.95	4.66E-02	LOC106576751	glutathione S-transferase kappa 1-like 2C transcript variant X1
XM_014168196.1	1.12	4.68E-02	LOC106583697	cytochrome c oxidase assembly protein COX14 homolog 2C transcript variant X1
XM_014134839.1	1.22	4.75E-02	mrps25	mitochondrial ribosomal protein S25
XM_014122877.1	0.92	4.79E-02	LOC106560233	uncharacterized LOC106560233 2C transcript variant X1
XM_014170114.1	1.13	4.79E-02	LOC106584630	ubiquitin-60S ribosomal protein L40
XM_014205335.1	-3.26	4.79E-02	LOC106607875	tumor necrosis factor receptor superfamily member 21-like
XM_014206408.1	-0.83	4.85E-02	LOC106608479	putative transcription factor Ovo-like 1
NM_001140361.1	0.48	4.91E-02	if4ea	Eukaryotic translation initiation factor 4E-1A
XM_014166598.1	1.26	4.91E-02	LOC106582956	28S ribosomal protein S16 2C mitochondrial-like
XM_014179703.1	0.69	4.91E-02	LOC106589571	ATP synthase mitochondrial F1 complex assembly factor 2-like 2C transcript variant X1
XM_014204144.1	0.95	4.91E-02	LOC106607307	coiled-coil domain-containing protein 43-like
XM_014204227.1	0.54	4.91E-02	LOC106607342	lipopolysaccharide-induced tumor necrosis factor-alpha factor homolog 2C transcript variant X1
NM_001139600.1	1.22	4.91E-02	rps29	ribosomal protein S29
NM_001141313.1	2.18	4.91E-02	tnni1	troponin I 2C slow skeletal muscle
NM_001252352.1	0.60	4.91E-02	xbp1	X-box binding protein 1

Table S5. Selected differentially expressed genes with known immune function following 24h exposure to LPS (20 µg/ml) in control (non-stressed) fish. In total, 25,469 genes were differentially regulated (FDR <0.05) by LPS exposure. Shading indicates up (red) or down (green) regulation.

Accession	Log2 Fold Change	FDR	Gene name	Gene description
NM_001141110.1	-2.88	3.61E-15	nkl	Antimicrobial peptide NK-lysin
XM_014129907.1	-4.54	1.23E-04	LOC106563926	antimicrobial peptide NK-lysin-like
XM_014207836.1	1.26	5.11E-19	LOC106609252	apoptosis facilitator Bcl-2-like protein 14_2C transcript variant X1
XM_014148534.1	-3.33	2.94E-03	LOC106573461	apoptosis-associated speck-like protein containing a CARD
XM_014185593.1	-1.07	1.00E-04	LOC106594220	apoptosis-inducing factor 1_2C mitochondrial-like
NM_001141505.1	-1.23	8.01E-07	apitd1	apoptosis-inducing_2C TAF9-like domain 1
XM_014211146.1	1.09	1.44E-05	LOC106611188	apoptosis-stimulating of p53 protein 1-like_2C transcript variant X1
XM_014187907.1	-3.52	6.73E-09	LOC106596637	B-cell antigen receptor complex-associated protein alpha chain-like_2C
XM_014155916.1	-1.35	8.30E-12	LOC106577630	B-cell CLL/lymphoma 6 member B protein-like_2C transcript variant X1
XM_014171355.1	2.36	8.69E-04	LOC106585288	B-cell CLL/lymphoma 7 protein family member A-like
XM_014155482.1	1.51	3.01E-11	LOC106577470	B-cell lymphoma 3 protein-like_2C transcript variant X1
XM_014206088.1	4.25	5.22E-09	LOC106608246	B-cell lymphoma/leukemia 11B-like_2C transcript variant X1
XM_014157075.1	4.29	6.98E-03	LOC106578359	B-cell receptor CD22-like_2C transcript variant X1
XM_014187472.1	-1.58	3.79E-13	LOC106596153	CASP8 and FADD-like apoptosis regulator
XM_014203779.1	1.03	4.17E-11	LOC106607148	caspase recruitment domain-containing protein 10-like
XM_014214559.1	-1.08	1.78E-12	LOC106612902	caspase-1-like
XM_014137997.1	2.98	3.19E-45	LOC106568012	caspase-3-like
XM_014160142.1	-1.51	3.87E-08	LOC106579851	C-C chemokine receptor type 4-like
XM_014160129.1	-1.26	4.73E-07	LOC106579844	C-C chemokine receptor type 5-like
XM_014160242.1	-1.18	2.86E-07	LOC106579893	C-C chemokine receptor type 5-like_2C transcript variant X1
XM_014176636.1	-1.21	1.64E-03	LOC106587975	C-C motif chemokine 13-like
XM_014128861.1	-1.99	5.01E-11	LOC106563358	C-C motif chemokine 19-like
XM_014141244.1	2.73	1.84E-23	LOC106569680	C-C motif chemokine 20-like
NM_001141267.2	-1.24	9.46E-07	ccl21	C-C motif chemokine 21
XM_014172601.1	-1.49	2.47E-05	LOC106585882	C-C motif chemokine 4-like
XM_014124357.1	-1.15	7.00E-04	LOC106560915	C-C motif chemokine 8-like
XM_014149455.1	1.54	2.87E-03	LOC106573945	CD109 antigen-like
XM_014209668.1	-2.93	1.61E-14	LOC106610349	CD209 antigen-like protein E
XM_014168155.1	2.33	7.30E-09	LOC106583660	CD276 antigen-like
XM_014175885.1	1.60	4.39E-04	LOC106587471	CD81 antigen-like
XM_014147496.1	1.09	2.03E-03	LOC106572911	CD82 antigen-like
XM_014163256.1	1.29	1.16E-04	LOC106581291	CD83 antigen-like
XM_014187383.1	-1.82	1.48E-03	LOC106596062	chemokine XC receptor 1-like
XM_014156674.1	1.08	2.19E-08	LOC106578085	chemokine-like receptor 1
XM_014186332.1	6.97	5.23E-04	LOC106594945	C-myc promoter-binding protein-like
XM_014178639.1	-1.16	1.01E-05	LOC106589014	C-Myc-binding protein-like_2C transcript variant X1
XM_014148242.1	1.86	2.45E-05	LOC106573304	complement C1q tumor necrosis factor-related protein 4-like
XM_014143850.1	-6.04	7.38E-03	LOC106571137	complement C1q-like protein 2
XM_014162291.1	2.07	5.40E-03	LOC106580826	complement C2-like
XM_014189274.1	1.13	1.37E-04	LOC106598222	complement C4-like
NM_001140784.1	5.82	1.10E-14	c1ql2	complement component 1_2C q subcomponent-like 2
NM_001146430.1	-2.36	5.41E-03	c8g	complement component 8_2C gamma polypeptide
XM_014134762.1	1.79	9.62E-15	LOC106566600	complement component C7-like
XM_014169804.1	-1.07	1.83E-07	LOC106584449	complement factor D-like
NM_001140872.1	-1.09	1.02E-03	cmc4	C-x(9)-C motif containing 4
XM_014152244.1	3.15	2.21E-07	LOC106575635	C-X-C chemokine receptor type 1-like
XM_014152259.1	4.73	2.40E-08	LOC106575644	C-X-C chemokine receptor type 2-like
XM_014157979.1	-1.01	4.30E-05	LOC106578808	C-X-C chemokine receptor type 3-like_2C transcript variant X1
XM_014164248.1	1.54	2.10E-15	LOC106581841	C-X-C chemokine receptor type 4-like
NM_001141028.1	-1.55	3.24E-07	cxl10	C-X-C motif chemokine 10

XM_014131006.1	1.61	4.38E-08	LOC106564720	C-X-C motif chemokine 11-like
XM_014172589.1	-2.06	3.76E-04	LOC106585872	C-X-C motif chemokine 13-like
XM_014205326.1	-1.12	9.64E-03	LOC106607873	C-X-C motif chemokine 13-like_2C transcript variant X1
NM_001140825.1	1.35	7.36E-03	cxl14	C-X-C motif chemokine 14
XM_014150420.1	1.88	3.32E-29	LOC106574504	cytokine receptor-like factor 1
XM_014151881.1	1.05	5.57E-07	LOC106575381	cytokine receptor-like factor 1_2C transcript variant X1
XM_014143589.1	1.91	6.16E-04	LOC106570976	death-associated protein kinase 3-like
XM_014155116.1	-1.23	3.34E-03	LOC106577211	DNA damage-inducible transcript 4 protein-like
XM_014212990.1	1.23	5.51E-09	LOC106612111	DNA damage-inducible transcript 4-like protein_2C
XM_014158346.1	-1.11	3.73E-13	dnajc9	DnaJ (Hsp40) homolog_2C subfamily C_2C member 9_2C
XM_014168282.1	2.55	3.54E-07	LOC106583738	dnaJ homolog subfamily B member 12-like
XM_014161119.1	-1.36	5.48E-05	LOC106580287	dnaJ homolog subfamily B member 5-like_2C transcript variant X1
XM_014129653.1	-2.54	2.03E-03	LOC106563796	dnaJ homolog subfamily C member 3-like
XM_014158372.1	1.07	1.22E-06	LOC106578994	HEAT repeat-containing protein 1-like_2C transcript variant X1
XM_014202722.1	5.84	6.76E-03	LOC106606490	heat shock 70 kDa protein 12A-like_2C transcript variant X1
XM_014186568.1	1.32	9.43E-03	LOC106595188	heat shock 70 kDa protein 14-like
XM_014192866.1	1.84	2.01E-06	LOC106601018	heat shock 70 kDa protein-like
XM_014214839.1	1.53	5.35E-29	LOC106613013	heat shock cognate 70 kDa protein-like_2C transcript variant X1
NM_001146651.1	1.48	6.19E-26	hsp7c	Heat shock cognate 71 kDa protein
XM_014215074.1	-5.73	1.40E-04	LOC106613119	heat shock protein beta-7-like
NM_001146578.1	-1.23	5.42E-06	hspb11	heat shock protein family B (small)_2C member 11
XM_014205881.1	-1.63	2.01E-06	LOC106608137	heat shock protein HSP 90-alpha 1
XM_014140001.1	-1.15	3.44E-03	LOC106569050	immunoglobulin iota chain-like
XM_014202524.1	1.72	5.56E-03	LOC106606374	immunoglobulin lambda-like polypeptide 1
XM_014126092.1	-1.33	7.84E-03	ighmbp2	immunoglobulin mu binding protein 2
XM_014126795.1	-1.34	5.25E-03	LOC106562145	immunoglobulin superfamily DCC subclass member 4-like_2C
XM_014215398.1	1.10	2.13E-05	LOC106613308	immunoglobulin superfamily member 10-like
XM_014213999.1	-1.87	1.31E-06	LOC106612643	immunoglobulin superfamily member 11-like_2C transcript variant X1
XM_014175399.1	-4.45	4.66E-04	LOC106587223	immunoglobulin superfamily member 1-like_2C transcript variant X1
XM_014162937.1	-2.62	9.39E-05	LOC106581125	immunoglobulin superfamily member 5-like
XM_014194077.1	1.00	4.94E-06	LOC106601731	immunoglobulin superfamily member 6-like
XM_014186408.1	1.06	4.18E-10	LOC106595027	immunoglobulin-like domain-containing receptor 1
XM_014151036.1	1.88	1.32E-03	LOC106574905	immunoglobulin-like domain-containing receptor 2
XM_014163747.1	4.62	9.12E-66	irg1	immunoresponsive 1 homolog (mouse)
NM_001123558.1	-1.27	9.95E-03	ifng	Interferon gamma
XM_014187639.1	-2.04	2.45E-03	LOC106596334	interferon a3-like_2C transcript variant X1
NM_001123710.1	-4.25	1.17E-05	ifna1	interferon alpha 1
NM_001252364.1	-1.03	9.66E-08	irf1-2	interferon regulatory factor 1 isoform 2
XM_014162109.1	1.08	2.74E-03	LOC106580746	interferon regulatory factor 2-binding protein 1-like_2C
XM_014145468.1	-2.01	8.62E-16	LOC106571913	interferon regulatory factor 4-like_2C transcript variant X1
XM_014133038.1	1.53	7.52E-15	LOC106565674	interferon regulatory factor 6-like_2C transcript variant X1
XM_014165670.1	-7.13	1.10E-06	LOC106582502	interferon-induced guanylate-binding protein 1-like
NM_001140400.1	-1.09	6.74E-10	ifi44	Interferon-induced protein 44
XM_014151635.1	-1.04	9.18E-03	LOC106575246	interferon-induced protein 44-like
XM_014167589.1	-1.51	1.98E-03	LOC106583433	interferon-induced protein 44-like_2C transcript variant X1
XM_014154814.1	-1.30	1.52E-04	LOC106577091	interferon-induced protein with tetratricopeptide repeats 1-like
XM_014152521.1	2.94	1.11E-13	il11	interleukin 11_2C transcript variant X1
XM_014137694.1	1.26	4.40E-08	il18bp	interleukin 18 binding protein
NM_001204895.1	-1.05	6.09E-03	il4/13a	interleukin 4/13A
XM_014143360.1	2.94	2.47E-27	LOC106570815	interleukin-1 beta-like
NM_001145420.1	3.03	4.10E-52	il1r2	interleukin-1 receptor type II
XM_014153887.1	1.05	3.16E-06	LOC106576643	interleukin-1 receptor-associated kinase 3-like
XM_014201821.1	2.95	4.21E-18	LOC106605846	interleukin-11-like
XM_014155985.1	2.05	5.73E-03	LOC106577668	interleukin-13 receptor subunit alpha-2-like
XM_014134958.1	2.87	6.45E-04	LOC106566684	interleukin-17 receptor B-like_2C transcript variant X1
XM_014134418.1	1.31	7.25E-03	LOC106566413	interleukin-17 receptor D-like

XM_014149154.1	2.02	8.83E-08	LOC106573803	interleukin-17C-like
XM_014152609.1	-1.02	3.10E-03	LOC106575893	interleukin-18 receptor accessory protein-like_2C transcript variant X1
XM_014127964.1	1.66	3.22E-03	LOC106562893	interleukin-6 receptor subunit beta-like_2C transcript variant X1
XM_014129604.1	1.71	3.79E-03	LOC106563758	interleukin-7 receptor subunit alpha-like_2C transcript variant X1
NM_001140775.1	-1.68	5.94E-12	cd37	Leukocyte antigen CD37
XM_014140842.1	1.10	1.13E-03	LOC106569467	leukocyte cell-derived chemotaxin 1-like_2C transcript variant X1
XM_014211962.1	1.58	4.12E-11	LOC106611589	leukocyte cell-derived chemotaxin-2-like_2C transcript variant X1
XM_014201687.1	1.47	5.54E-08	LOC106605761	leukocyte receptor cluster member 8 homolog_2C transcript variant X1
XM_014201694.1	1.13	1.10E-22	LOC106605766	leukocyte receptor cluster member 9-like_2C transcript variant X1
NM_001159373.1	-1.12	1.72E-04	ll	leukolectin protein
NM_001140748.1	2.30	3.15E-05	lt4r1	Leukotriene B4 receptor 1
XM_014191684.1	1.89	2.40E-11	LOC106600382	leukotriene B4 receptor 1-like_2C transcript variant X1
XM_014153881.1	-1.06	9.11E-05	LOC106576634	lymphocyte activation gene 3 protein-like
XM_014202496.1	-1.54	1.99E-12	LOC106606339	lymphocyte-specific helicase-like_2C transcript variant X1
NM_001141513.1	-1.60	1.07E-09	topk	Lymphokine-activated killer T-cell-originated protein kinase homolog
NM_001141422.2	1.76	8.53E-05	mip2a	Macrophage inflammatory protein 2-alpha
NM_001141547.1	-1.07	8.85E-06	mif	macrophage migration inhibitory factor
XM_014164327.1	-2.94	3.58E-17	LOC106581890	major histocompatibility complex class I-related gene protein-like
XM_014188250.1	-2.92	1.79E-04	LOC106597015	major histocompatibility complex class I-related gene protein-like_2C
XM_014182254.1	9.14	4.89E-07	LOC106591067	mucin-12-like
XM_014151948.1	3.55	1.03E-04	LOC106575448	mucin-17-like
XM_014215536.1	4.45	2.73E-03	LOC106613376	mucin-19-like
XM_014177637.1	7.88	6.73E-21	LOC106588522	mucin-2-like
XM_014187672.1	3.21	2.05E-12	LOC106596373	mucin-2-like
XM_014177096.1	4.52	1.50E-04	LOC106588256	mucin-2-like
XM_014195406.1	11.15	7.77E-61	LOC106602652	mucin-5AC-like
XM_014191480.1	6.92	3.52E-29	LOC106600148	mucin-5AC-like
XM_014175874.1	2.56	3.80E-13	LOC106587462	mucin-5B-like
XM_014123270.1	1.74	1.14E-04	LOC106560392	mucin-7-like_2C transcript variant X1
XM_014182612.1	3.02	8.72E-03	LOC106591388	neutrophil cytosol factor 4-like
XM_014183791.1	1.37	8.57E-08	LOC106592447	NF-kappa-B essential modulator-like
XM_014204687.1	1.02	1.95E-14	LOC106607598	NF-kappa-B inhibitor alpha-like
XM_014214975.1	1.91	2.52E-04	nos2	nitric oxide synthase 2_2C inducible_2C transcript variant X1
XM_014164090.1	-1.10	2.52E-04	nostrin	nitric oxide synthase trafficking
XM_014159943.1	3.93	1.37E-04	LOC106579776	NK-tumor recognition protein-like
XM_014130660.1	1.14	9.88E-06	LOC106564532	nuclear factor interleukin-3-regulated protein-like_2C transcript
XM_014175275.1	1.20	2.27E-07	LOC106587185	nuclear factor of activated T-cells 5-like_2C transcript variant X1
XM_014177036.1	1.07	2.85E-06	LOC106588230	nuclear factor of activated T-cells_2C cytoplasmic 1-like_2C
XM_014177007.1	-1.14	7.38E-06	LOC106588224	programmed cell death 1 ligand 1-like
XM_014185045.1	2.43	7.28E-03	LOC106593672	programmed cell death 6-interacting protein-like
XM_014183194.1	1.36	1.40E-09	LOC106591927	suppressor of cytokine signaling 1-like
XM_014194350.1	1.96	1.38E-15	LOC106601915	suppressor of cytokine signaling 3-like
XM_014192483.1	2.10	5.81E-04	LOC106600801	suppressor of cytokine signaling 7-like_2C transcript variant X1
XM_014129570.1	-1.57	3.82E-07	LOC106563739	T-cell surface antigen CD2-like_2C transcript variant X1
XM_014189050.1	-1.59	1.16E-08	LOC106597940	T-cell surface glycoprotein CD1c-like_2C transcript variant X1
XM_014162423.1	1.95	6.44E-03	LOC106580890	T-cell surface glycoprotein CD3 delta chain-like
XM_014199173.1	-2.39	2.06E-04	LOC106604508	T-cell surface glycoprotein CD8 beta chain-like
XM_014142947.1	-1.06	7.33E-09	LOC106570527	TLR4 interactor with leucine rich repeats-like
XM_014178347.1	-1.02	7.47E-07	LOC106588859	TLR4 interactor with leucine rich repeats-like
XM_014137409.1	-1.77	1.49E-03	LOC106567744	TNF receptor-associated factor 2-like
XM_014163086.1	1.54	9.68E-04	LOC106581191	TNF receptor-associated factor 4-like
XM_014178938.1	-1.32	2.87E-05	LOC106589219	toll-like receptor 13
XM_014199800.1	1.16	2.37E-07	LOC106604777	toll-like receptor 6
XM_014157098.1	1.01	1.03E-12	LOC106578374	TRAF2 and NCK-interacting protein kinase-like_2C transcript variant X1
XM_014139936.1	3.37	3.19E-38	LOC106569003	transforming growth factor beta-1-like
NM_001123617.1	1.08	3.18E-03	LOC100136509	tumor necrosis factor alpha

NM_001123590.1	3.05	1.99E-09	tnf-alpha-2	tumor necrosis factor alpha-2 precursor
XM_014181607.1	-1.41	1.70E-19	LOC106590519	tumor necrosis factor ligand superfamily member 10-like_2C
XM_014206883.1	-1.88	8.28E-18	LOC106608761	tumor necrosis factor ligand superfamily member 12-like
XM_014170639.1	1.86	5.44E-28	LOC106584955	tumor necrosis factor receptor superfamily member 10B-like_2C
XM_014177272.1	1.68	4.10E-11	LOC106588364	tumor necrosis factor receptor superfamily member 11B-like
XM_014196829.1	-1.21	1.25E-03	LOC106603311	tumor necrosis factor receptor superfamily member 19-like
XM_014163439.1	3.29	1.19E-23	LOC106581397	tumor necrosis factor receptor superfamily member 19L-like
XM_014205335.1	3.66	5.64E-08	LOC106607875	tumor necrosis factor receptor superfamily member 21-like
XM_014133628.1	4.89	2.27E-21	LOC106565937	tumor necrosis factor receptor superfamily member 21-like_2C
XM_014153851.1	4.05	2.33E-11	LOC106576603	tumor necrosis factor receptor superfamily member 5-like
XM_014146103.1	3.35	8.54E-34	LOC106572181	tumor necrosis factor receptor superfamily member 6B-like
XM_014146497.1	2.20	2.39E-44	LOC106572390	tumor necrosis factor receptor superfamily member 9-like_2C
XM_014136451.1	3.00	1.42E-32	tnfrsf6b	tumor necrosis factor receptor superfamily_2C member 6b_2C
XM_014182070.1	3.48	6.91E-05	LOC106590894	tumor protein D52-like
XM_014134677.1	1.11	8.94E-09	tp53inp2	tumor protein p53 inducible nuclear protein 2_2C transcript variant X1
XM_014211497.1	-1.15	1.21E-05	tp53i3	tumor protein p53 inducible protein 3_2C transcript variant X1
XM_014176373.1	1.04	2.84E-04	LOC106587750	tumor suppressor p53-binding protein 1-like
XM_014206242.1	4.38	9.61E-03	myb	v-myb avian myeloblastosis viral oncogene homolog
XM_014166334.1	-1.28	1.98E-09	mybl2	v-myb avian myeloblastosis viral oncogene homolog-like 2_2C transcript
XM_014215119.1	3.06	5.92E-05	LOC106613145	von Willebrand factor A domain-containing protein 5A-like_2C transcript
XM_014209801.1	2.24	4.14E-14	LOC106610433	von Willebrand factor A domain-containing protein 7-like
XM_014190593.1	-1.05	1.79E-14	LOC106599384	von Willebrand factor C domain-containing protein 2-like
XM_014151598.1	4.75	3.19E-08	LOC106575228	von Willebrand factor D and EGF domain-containing protein-like
XM_014126136.1	1.14	3.73E-03	LOC106561866	von Willebrand factor-like
XM_014159196.1	1.54	3.74E-09	LOC106579350	Wilms tumor protein 1-interacting protein-like_2C transcript variant X1

Table S6. Summary of statistics for RRBS libraries

Group	Sample ID	Single raw reads (millions)	QC reads (millions)	Total alignment (%)	Unique alignment (%)	CpG methylation (%)	CHG methylation (%)	CHH methylation (%)	Bisulphite conversion efficiency (%)	Inappropriate conversion (%)
Control	15	57.91	57.84	91.46	43.29	74.68	0.38	0.28	99.80	2.50
Control	16	87.99	87.73	89.88	43.22	73.96	0.48	0.36	99.60	2.60
Control	17	102.56	102.42	92.60	44.52	73.60	0.47	0.36	99.60	2.00
Control	18	65.43	65.36	92.77	44.95	74.42	0.46	0.37	99.60	2.40
Control	40	77.47	77.36	87.12	42.58	77.05	0.40	0.30	99.80	2.30
Control	45	33.72	33.56	91.77	43.15	74.61	0.48	0.36	99.70	2.60
Control	46	49.49	49.45	92.76	44.93	75.58	0.47	0.37	99.70	1.70
Control	47	60.37	60.36	90.15	43.40	75.08	0.37	0.28	99.70	1.90
Control	48	46.43	46.30	89.52	42.66	74.01	0.39	0.29	99.80	2.40
Cold shock	25	61.67	61.63	91.65	44.19	74.84	0.47	0.37	99.60	2.00
Cold shock	26	59.70	59.60	92.36	44.20	76.02	0.47	0.37	99.70	1.80
Cold shock	27	74.88	74.79	92.30	43.48	76.42	0.48	0.36	99.60	1.90
Cold shock	28	48.04	47.90	91.66	42.44	76.11	0.49	0.36	99.70	1.60
Cold shock	55	51.63	51.62	90.75	44.58	72.96	0.37	0.27	99.70	2.00
Cold shock	56	50.06	50.06	90.34	44.06	72.03	0.37	0.27	99.70	1.40
Cold shock	57	55.09	54.90	90.75	43.03	72.40	0.39	0.29	99.90	1.50
Cold shock	58	70.79	70.78	90.06	43.88	71.54	0.37	0.27	99.70	3.00
Substrate stress	9	67.72	67.66	91.17	43.26	78.18	0.40	0.29	99.80	2.20
Substrate stress	10	74.09	72.91	88.24	40.43	73.81	0.48	0.36	99.60	2.00
Substrate stress	35	64.73	64.70	92.31	44.51	78.07	0.47	0.37	99.70	1.60
Substrate stress	36	78.69	78.66	87.41	44.22	72.72	0.37	0.28	99.70	1.90
Substrate stress	37	62.45	62.32	87.82	42.05	74.75	0.39	0.30	99.80	2.60
Substrate stress	38	63.47	63.46	89.08	44.35	71.80	0.36	0.27	99.80	3.10
Substrate stress	39	72.97	72.80	90.44	42.11	75.24	0.49	0.37	99.60	2.00
Mean		*63.73*	*63.59*	*90.42*	*43.39*	*74.46*	*0.42*	*0.32*	*99.71*	*2.04*

Table S7a. Genes with altered baseline expression and p.promoter methylation in acutely stressed fish

Gene	Gene description	Δ Methylation (%)	Δ Expression (Log2 FC)
LOC106577598	excitatory amino acid transporter 3-like	-7.17	3.18
LOC106563595	protein shisa-2 homolog_2C transcript variant X1	-6.11	2.74
LOC106606008	growth hormone secretagogue receptor type 1-like	-6.24	2.42
LOC106596092	uncharacterized ncRNA	-11.77	1.99
LOC106601990	dexamethasone-induced Ras-related protein 1-like	-21.69	1.99
LOC106568995	serotransferrin-1-like	-5.11	1.81
LOC106611560	glutamate receptor 2-like_2C transcript variant X1	-6.45	1.77
LOC106610943	XK-related protein 6-like_2C transcript variant X1	-19.97	1.67
LOC106578303	serine/threonine-protein kinase BRSK2-like_2C variant X1	-17.09	1.45
LOC106562184	uncharacterized ncRNA	-6.70	1.35
LOC106610664	peptide methionine sulfoxide reductase MsrA 1-like	-8.94	1.31
LOC106600757	uncharacterized	-5.92	1.22
LOC106561738	sperm acrosome membrane-associated protein 4-like	-6.78	1.19
LOC106579386	uncharacterized	-5.59	1.12

Gene	Gene description	Δ Methylation (%)	Δ Expression (Log2 FC)
LOC106563054	V-type proton ATPase subunit B_2C brain isoform	7.16	1.11
LOC106565116	BTB/POZ domain-containing protein KCTD20-l2C X1	9.79	1.06
LOC106569644	YEATS domain-containing protein 2-like_2C transcript X1	13.51	1.88
LOC106573335	cilia- and flagella-associated protein 44-like_2C transcript	5.52	1.39
cxl13	C-X-C motif chemokine 13	5.89	1.13
nkx2-6	NK2 homeobox 6%2C transcript variant X1	5.09	1.502715936

Gene	Gene description	Δ Methylation (%)	Δ Expression (Log2 FC)
hepc1	Hepcidin-1	-7.45	-1.88
LOC106568783	calcium-activated potassium channel subunit beta-3-like	-6.23	-1.29
LOC106600637	protein shisa-3 homolog	-16.37	-1.58
LOC106569906	uncharacterized LOC106569906	-9.73	-4.71
LOC106572843	tetraspan membrane protein of hair cell stereocilia	-6.65	-2.49
LOC106581468	extracellular calcium-sensing receptor-like	-8.61	-4.48
LOC106602114	tyrosine-protein phosphatase 13	-7.11	-1.12
LOC106612219	BTB/POZ domain-containing protein KCTD16-like	-6.31	-2.88

Gene	Gene description	Δ Methylation (%)	Δ Expression (Log2 FC)
LOC106580260	natterin-3-like	5.19	-5.55
trp-ii	trypsin II	11.89	-5.32
LOC106602273	TATA box-binding protein-like protein 2	8.54	-3.34
LOC106566573	opsin-5-like_2C transcript variant X1	5.95	-3.14
LOC106586110	uncharacterized ncRNA	5.65	-3.02
LOC106575957	protein Wnt-7b-like	9.39	-1.77
LOC106582036	tryptophan--tRNA ligase, mitochondrial-like	6.06	-1.60
epo	erythropoietin_2C transcript variant X1	5.49	-1.44
LOC106580238	uncharacterized	8.66	-1.25
LOC106586743	uncharacterized ncRNA	5.23	-1.25
LOC106608787	ATP-binding cassette sub-family A member 1-like_2C	8.21	-1.21
LOC106613793	uncharacterized ncRNA	6.15	-1.00

Table S7b. Genes with altered baseline expression and p.promoter methylation in chronically stressed fish

Gene	Gene description	Δ Methylation (%)	Δ Expression (Log2 FC)
LOC106561413	transcription factor RFX4-like_2C transcript variant X1	-5.42	1.52
LOC106563595	protein shisa-2 homolog_2C transcript variant X1	-10.71	2.21
LOC106566683	uncharacterized ncRNA	-7.20	1.37
LOC106569644	YEATS domain-containing protein 2-like_2C transcript X1	-11.25	1.46
LOC106573011	Uncharacterized	-14.81	1.20
LOC106576054	aldose reductase-like	-12.99	1.16
LOC106577897	plasminogen activator inhibitor 1-like	-7.75	1.54
LOC106577954	uncharacterized ncRNA	-6.21	3.15
LOC106583227	transcription factor Sp5-like	-7.89	1.11
LOC106584559	sperm acrosome membrane-associated protein 4-like_2C	-8.06	3.21
LOC106585649	complement C1q-like protein 2	-5.62	1.66
LOC106585892	piggyBac transposable element-derived protein 4-like_2C	-5.25	1.66
LOC106590765	potassium voltage-gated channel subfamily H member 7-2C	-5.46	1.07
LOC106602848	vitronectin-like	-7.75	1.09
LOC106604908	rod cGMP-specific 3',5'-cyclic phosphodiesterase alpha-like	-9.50	1.35
LOC106606455	cytochrome P450 2K3-like	-12.30	1.33
LOC106608067	serine/threonine-protein kinase PAK 7 pseudogene	-6.60	2.02
LOC106608776	E3 ubiquitin-protein ligase KEG-like	-11.09	1.21
slc5a12	solute carrier family 5 (sodium/monocarboxylate) 2C	-5.53	1.44

Gene	Gene description	Δ Methylation (%)	Δ Expression (Log2 FC)
ier3	immediate early response 3	9.31	1.07
LOC106561977	carbonic anhydrase 7-like_2C transcript variant X1	11.78	1.35
LOC106566573	opsin-5-like_2C transcript variant X1	9.44	1.52
LOC106592616	serine incorporator 5-like	9.850638875	1.182271959
LOC106593408	myosin heavy chain%2C fast skeletal muscle-like	8.4408575	1.215999959
LOC106613765	cyclic AMP-responsive element-binding protein 3-like protein 3-B	14.88	1.39
LOC106613773	zinc finger and BTB domain-containing protein 7A-like_2C X2	9.60	2.36

Gene	Gene description	Δ Methylation (%)	Δ Expression (Log2 FC)
LOC106560428	excitatory amino acid transporter 5-like	-8.40	-6.05
LOC106581468	extracellular calcium-sensing receptor-like	-6.94	-3.71
LOC106582596	olfactory receptor 51T1-like	-5.41	-11.84
LOC106586042	zinc finger protein ZIC 2-like	-7.06	-2.09
LOC106587141	adhesion G-protein coupled receptor G5-like%2C transcript	-10.42886	-1.074079782
LOC106592617	relaxin-3 receptor 1-like	-14.53788125	-2.460225901
LOC106602114	tyrosine-protein phosphatase 13	-5.12	-1.30
mcmdc2	minichromosome maintenance domain containing 2_2C	-12.89	-1.92

Gene	Gene description	Δ Methylation (%)	Δ Expression (Log2 FC)
LOC106560954	uncharacterized	8.37	-2.64
LOC106561337	DNA polymerase subunit gamma-1-like	6.32	-2.01
LOC106561347	uncharacterized ncRNA	12.56	-1.23
LOC106561447	cyclic nucleotide-gated cation channel beta-1-like_2C transcript t	5.63	-5.54
LOC106561738	sperm acrosome membrane-associated protein 4-like	10.38	-14.29
LOC106563054	V-type proton ATPase subunit B_2C brain isoform	6.88	-1.25
LOC106565359	sodium- and chloride-dependent GABA transporter 2-like	16.26	-1.03
LOC106571889	pro-thyrotropin-releasing hormone-A-like	5.29	-4.51
LOC106572438	membrane-assoc. guanylate kinase2C WW & PDZ domain	5.67	-2.33
LOC106575957	protein Wnt-7b-like	9.21	-4.71
LOC106579674	uncharacterized ncRNA	6.13	-1.07
LOC106588148	protein S100-A1-like_2C transcript variant X1	5.87	-1.11
LOC106593573	serine protease HTRA3 pseudogene	7.82	-3.73
LOC106601301	cilia- and flagella-associated protein 52-like	5.13	-2.83
LOC106605020	uncharacterized LOC106605020	6.40	-3.58
LOC106609265	ankyrin repeat and EF-hand domain-containing protein 1-like	5.10	-1.20
LOC106611494	multidrug resistance-associated protein 5-like	8.22	-2.03
trp-ii	trypsin II	5.31	-6.36

Table S8a. Genes with altered baseline expression and gene body methylation in acutely stressed fish

Gene	Gene description	Δ Methylation (%)	Δ Expression (Log2 FC)
LOC106565290	E3 ubiquitin-protein ligase RNF182-like	-5.17	4.01
LOC106570817	amyloid beta A4 precursor protein-binding family A	-6.35	1.17
LOC106573771	uncharacterized ncRNA	-7.19	2.49
LOC106576321	synaptotagmin-10-like_2C transcript variant X1	-5.57	1.71
LOC106581750	N-chimaerin-like	-5.90	1.06
LOC106581805	sodium-driven chloride bicarbonate exchanger-like_2C X1	-11.43	1.07
LOC106587310	uncharacterized ncRNA	-6.87	2.63
LOC106589807	muscular LMNA-interacting protein-like_2C transcript variant X3	-9.40	1.26
LOC106599779	voltage-dependent R-type calcium channel subunit alpha-1E-2C	-5.87	2.44
LOC106604113	netrin receptor UNC5A-like	-5.65	3.04
LOC106609476	protein FAM19A2-like_2C transcript variant X1	-6.31	1.12
LOC106610610	suppressor of cytokine signaling 4-like	-5.29	2.00
LOC106611538	BTB/POZ domain-containing protein KCTD8-like	-9.86	1.70
LOC106611781	nocturnin-like	-5.38	4.11
LOC106613542	alpha-N-acetylgalactosaminide alpha-2_2C6-sialyltransferase 5	-14.60	1.12

Gene	Gene description	Δ Methylation (%)	Δ Expression (Log2 FC)
LOC106560522	protein FAM183A-like	12.35	1.21
LOC106561437	nuclear pore complex protein Nup205-like	6.40	1.05
LOC106562934	Uncharacterized	6.86	2.05
LOC106564664	septin-9-like_2C transcript variant X1	14.47	1.16
LOC106569543	K/Na hyperpolarization-activated cyclic nucleotide-gated channel	5.64	1.23
LOC106571577	homeobox protein OTX2-like	7.67	1.65
LOC106572438	guanylate kinase_2C WW and PDZ domain-containing	5.02	4.08
LOC106573253	protein HOS4-like	5.15	1.21
LOC106575944	beta-galactoside-binding lectin-like	6.97	1.24
LOC106578851	heparan sulfate glucosamine 3-O-sulfotransferase 3B1-like_2C	7.99	1.78
LOC106580152	uncharacterized ncRNA	10.50	2.75
LOC106581793	beta-1_2C3-galactosyltransferase 1-like_2C transcript X1	5.76	1.90
LOC106586981	cysteine and glycine-rich protein 3-like	6.03	4.35
LOC106591735	T-box transcription factor TBX15-like	5.28	1.09
LOC106603892	prefoldin subunit 1-like	8.68	1.10
LOC106607511	MAGUK p55 subfamily member 3-like_2C transcript variant	8.95	1.16

Gene	Gene description	Δ Methylation (%)	Δ Expression (Log2 FC)
fat1	FAT atypical cadherin 1	-5.49	-1.23
LOC106563594	wiskott-Aldrich syndrome protein family member 3-like_2C	-6.78	-3.79
LOC106567517	solute carrier family 22 member 4-like	-5.06	-1.49
LOC106568191	uncharacterized ncRNA	-12.63	-1.65
LOC106568828	WD repeat-containing protein 37-like_2C transcript variant X1	-5.89	-1.52
LOC106569225	ankyrin repeat domain-containing protein 33B-like	-8.97	-3.11
LOC106570740	CUB and sushi domain-containing protein 1-like	-8.70	-1.28
LOC106573002	filamin-A-like	-7.32	-4.54
LOC106577486	cholecystokinin receptor type A-like	-5.40	-2.33
LOC106578039	5-hydroxytryptamine receptor 2C-like	-6.90	-1.02
LOC106579994	G patch domain-containing protein 8-like_2C transcript X1	-8.33	-1.46
LOC106580671	ATP-binding cassette sub-family G member 1-like	-6.32	-3.12
LOC106587255	uncharacterized ncRNA	-13.93	-1.21
LOC106587502	rho-related GTP-binding protein RhoU-like_2C transcript X1	-5.63	-1.79
LOC106590643	noelin-3-like	-6.59	-3.91
opn4x1b2	melanopsin	-6.79	-1.62

Gene	Gene description	Δ Methylation (%)	Δ Expression (Log2 FC)
LOC106561337	DNA polymerase subunit gamma-1-like	10.20	-2.01
LOC106563547	rod cGMP-specific 3'_2C5'-cyclic phosphodiesterase subunit β	10.94	-1.35
LOC106567739	ras-like protein family member 10B	6.50	-1.60
LOC106568063	WSC domain-containing protein 1-like	7.94	-2.66
LOC106568744	major facilitator superfamily domain-containing protein 8-like	6.41	-1.56
LOC106575141	GDP-Man:Man(3)GlcNAc(2)-PP-Dol mannosyltransferase	5.50	-2.19
LOC106575957	protein Wnt-7b-like	8.67	-1.77
LOC106577438	formin-2-like	5.32	-2.52
LOC106578999	retinol-binding protein 3-like	5.38	-1.06
LOC106580238	Uncharacterized	7.86	-1.25
LOC106607611	Uncharacterized	5.47	-1.23

Table S8b. Genes with altered baseline expression and gene body methylation in chronically stressed fish

Gene	Gene description	Δ Methylation (%)	Δ Expression (Log2 FC)
fgf13	fibroblast growth factor 13_2C transcript variant X1	-6.18	1.90
igfbp-2b2	insulin-like growth factor binding protein 2 paralog	-5.12	1.11
LOC106560781	uncharacterized ncRNA	-5.74	1.27
LOC106580950	P2Y purinoceptor 3-like	-12.67	2.03
LOC106581982	UDP-glucuronic acid decarboxylase 1-like_2C transcript X1	-5.27	2.59
LOC106608776	E3 ubiquitin-protein ligase KEG-like	-10.37	1.21
LOC106610951	neuroblast differentiation-associated protein AHNAK-like_2C X2	-6.26	1.23
LOC106613542	alpha-N-acetylgalactosaminide alpha-2_2C6-sialyltransferase 5	-13.29	1.06

Gene	Gene description	Δ Methylation (%)	Δ Expression (Log2 FC)
LOC106560893	tyrosine aminotransferase-like_2C transcript variant X1	7.94	1.20
LOC106568744	major facilitator superfamily domain-containing protein 8-like	5.36	2.38
LOC106568787	protein sidekick-2-like	6.19	1.00
LOC106580152	uncharacterized ncRNA	12.13	1.76
LOC106581508	amine sulfotransferase-like	11.25	1.07
LOC106599513	neuropilin-1a-like	6.04	1.85
LOC106599523	paired box protein Pax-3-like_2C transcript variant X1	16.72	1.35
LOC106603484	proline-rich protein 7-like_2C transcript variant X1	7.68	1.53
LOC106603687	heparin cofactor 2-like_2C transcript variant X1	5.43	1.23
LOC106607511	MAGUK p55 subfamily member 3-like_2C transcript variant	10.14	1.18
LOC106610780	mu-type opioid receptor-like_2C transcript variant X1	7.00	2.05
slc6a11	solute carrier family 6 (neurotransmitter transporter)_2C member	5.03	2.08

Gene	Gene description	Δ Methylation (%)	Δ Expression (Log2 FC)
cnbd1	cyclic nucleotide binding domain containing 1_2C transcript	-7.72	-1.68
LOC106563594	wiskott-Aldrich syndrome protein family member 3-like_2C	-11.39	-4.88
LOC106568431	uncharacterized ncRNA	-5.70	-5.51
LOC106582596	olfactory receptor 51T1-like	-5.59	-11.84
LOC106584188	BTB/POZ domain-containing protein KCTD1-like_2C X1	-9.48	-5.38
LOC106594335	zinc finger protein 883-like	-5.50	-1.48
LOC106600100	dynein heavy chain 11_2C axonemal-like	-5.32	-4.43
LOC106604259	actin-binding LIM protein 3-like	-6.64	-3.84
LOC106610190	uncharacterized ncRNA	-6.64	-1.14
LOC106610691	interphotoreceptor matrix proteoglycan 1-like_2C transcript X1	-5.12	-4.62
LOC106613873	leucine-rich repeat-containing protein 24-like	-5.62	-1.95
rl23a	60S ribosomal protein L23a	-5.38	-1.02

Gene	Gene description	Δ Methylation (%)	Δ Expression (Log2 FC)
LOC106560971	transmembrane protein 117-like	5.05	-1.65
LOC106562934	uncharacterized	5.33	-1.54
LOC106565685	leucine-rich repeat and fibronectin type-III protein	5.43	-1.29
LOC106567505	gamma-aminobutyric acid receptor subunit alpha-1-like_2C	11.30	-2.18
LOC106568087	uncharacterized ncRNA	5.57	-2.05
LOC106571955	kinesin heavy chain isoform 5A-like_2C transcript variant	5.02	-1.08
LOC106574564	fidgetin-like_2C transcript variant X1	5.31	-1.21
LOC106575141	GDP-Man:Man(3)GlcNAc(2)-PP-Dol -1,2-mannosyltransferase	5.82	-1.56
LOC106575586	uncharacterized ncRNA	5.82	-2.16
LOC106575957	protein Wnt-7b-like	8.99	-4.71
LOC106577813	sushi_2C von Willebrand factor type A_2C EGF	10.24	-1.02
LOC106580233	kinesin-like protein KIF27	5.84	-4.15
LOC106581393	glutamate receptor 4-like_2C transcript variant X1	5.52	-1.20
LOC106583727	metabotropic glutamate receptor 7-like	5.22	-4.72
LOC106585150	uncharacterized ncRNA	5.26	-3.96
LOC106586981	cysteine and glycine-rich protein 3-like	9.78	-2.81
LOC106605150	uncharacterized	13.97	-1.33
LOC106606219	regulator of G-protein signaling 9-like_2C transcript variant	7.63	-1.27
LOC106608697	nuclear factor 7_2C brain-like	9.82	-1.07
LOC106611369	neurexin-3a-beta-like	6.00	-1.81
scn2b	sodium channel_2C voltage gated_2C type II beta	6.50	-1.53

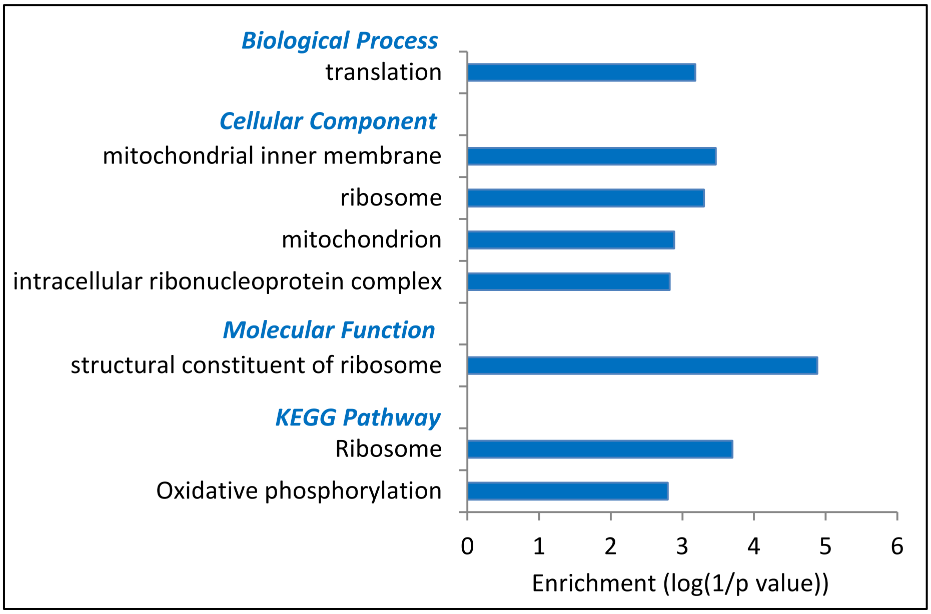

Figure S1. Gene Ontology Direct Terms and KEGG pathways significantly over-represented amongst the list of 206 differentially expressed genes identified in chronically-stressed fish compared to the control group (q<0.05).

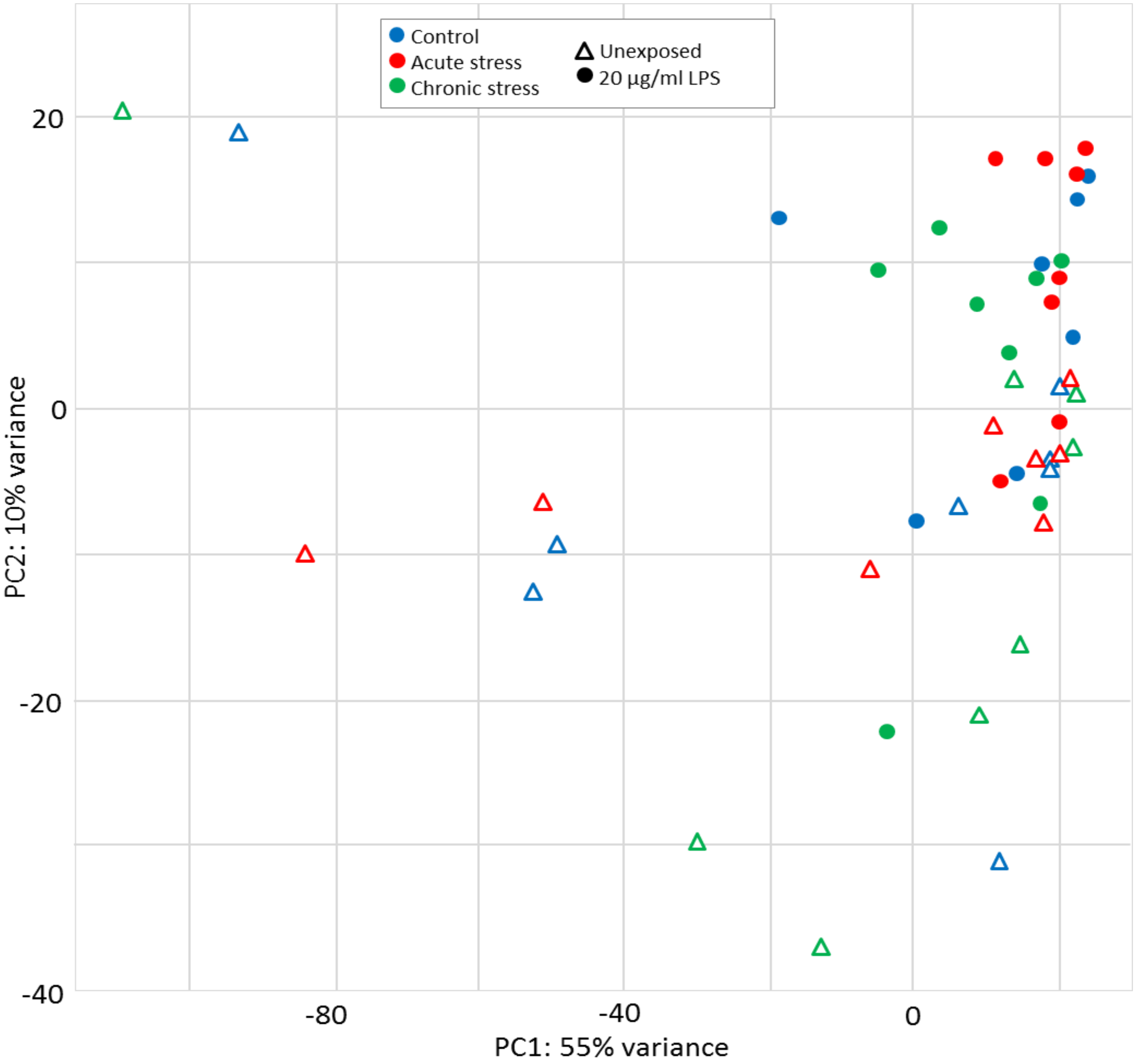

Figure S2. PCA illustrating the effect of exposure to 20 µg/ml LPS on the entire gill transcriptome (78,229 putative loci) of fish from all stress treatment groups.

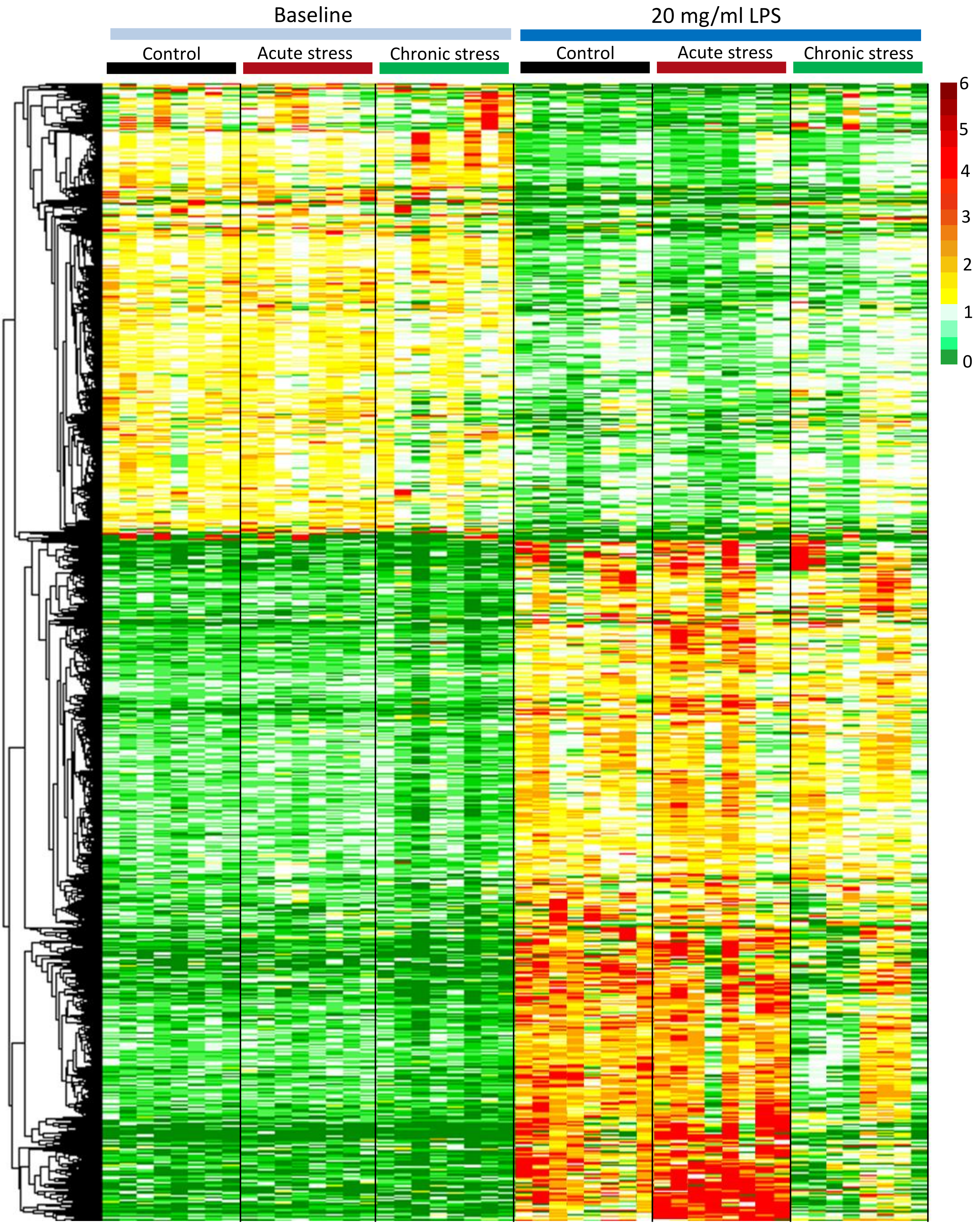

Figure S3. Heat map illustrating the expression level of all genes which were significantly regulated following exposure to 20 µg/ml LPS for 24h in the non-stressed control fish (>2 FC and FDR <0.01). Data presented are read counts for each individual normalised by library size, and by mean expression for each gene. Hierarchical clustering was performed using an Euclidian distance metric.

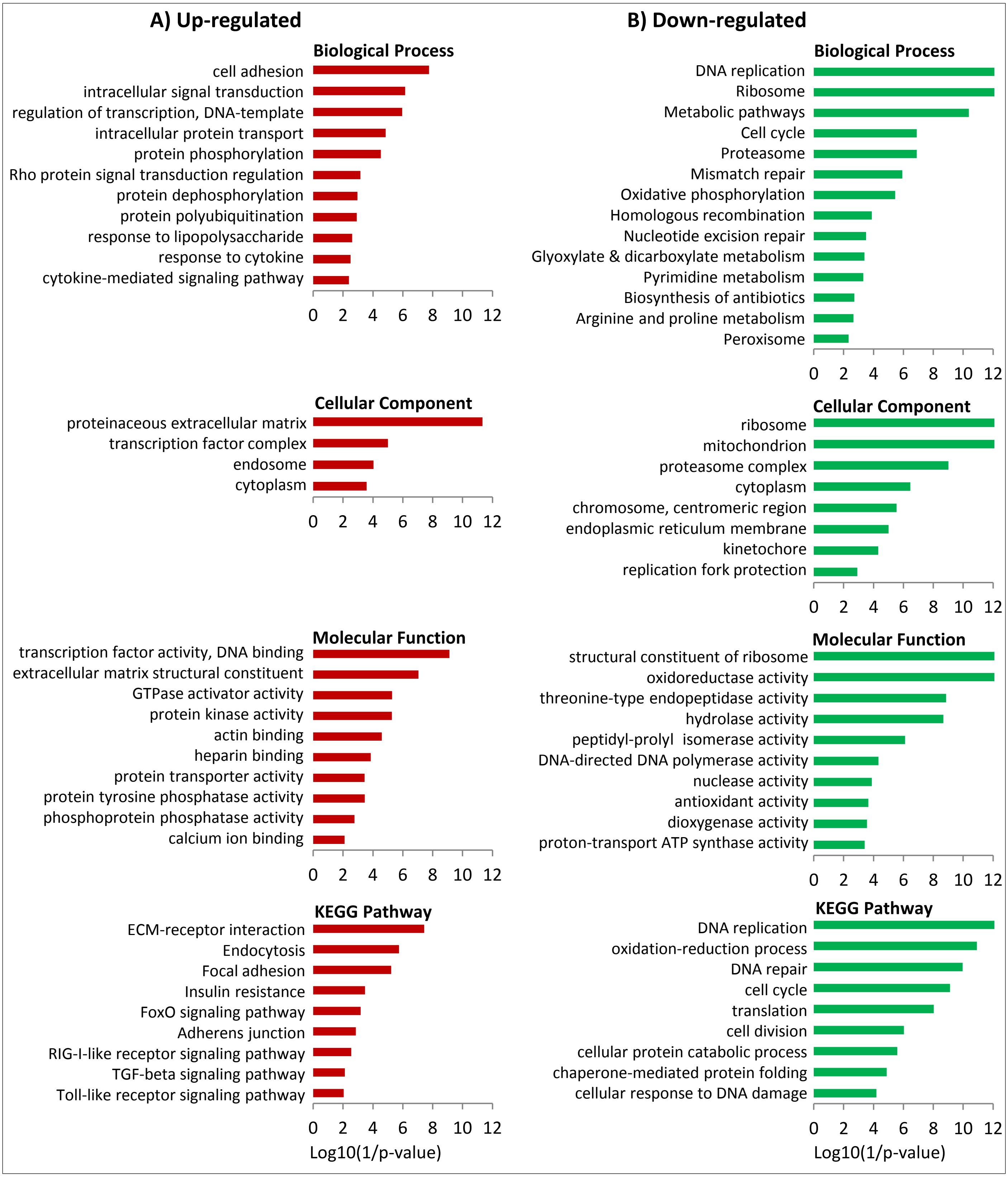

Figure S4. Enriched Gene Ontology Terms amongst A) up-regulated genes and B) down-regulated genes following exposure to 20 µg/ml LPS (for the non-stressed control group). Only enriched terms with $q < 0.05$ are displayed.

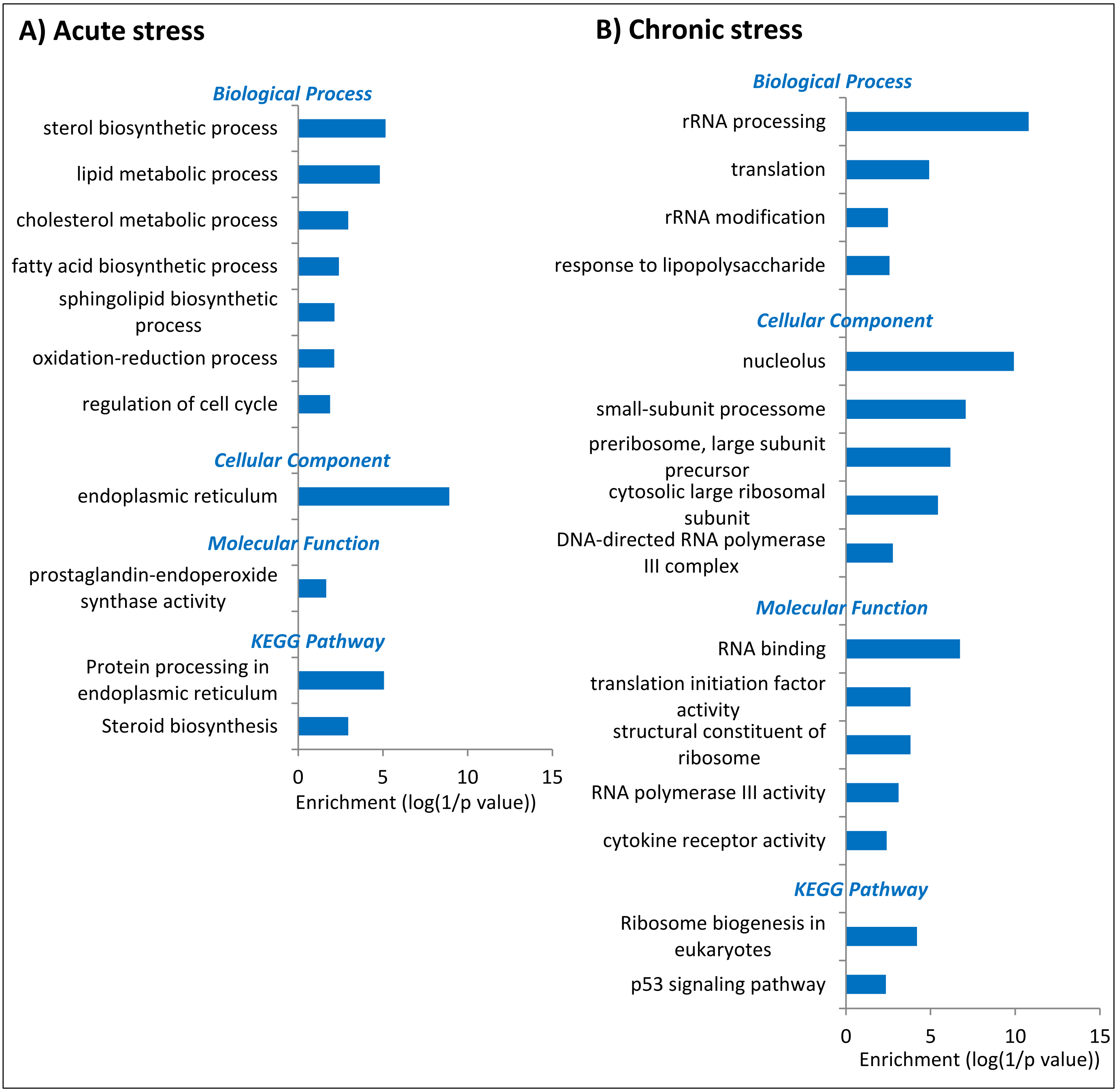

Figure S5. Gene Ontology Terms significantly over-represented (q<0.05) amongst the genes for which a significant interaction was identified between transcriptional response to LPS and A) acute stress or B) chronic stress.

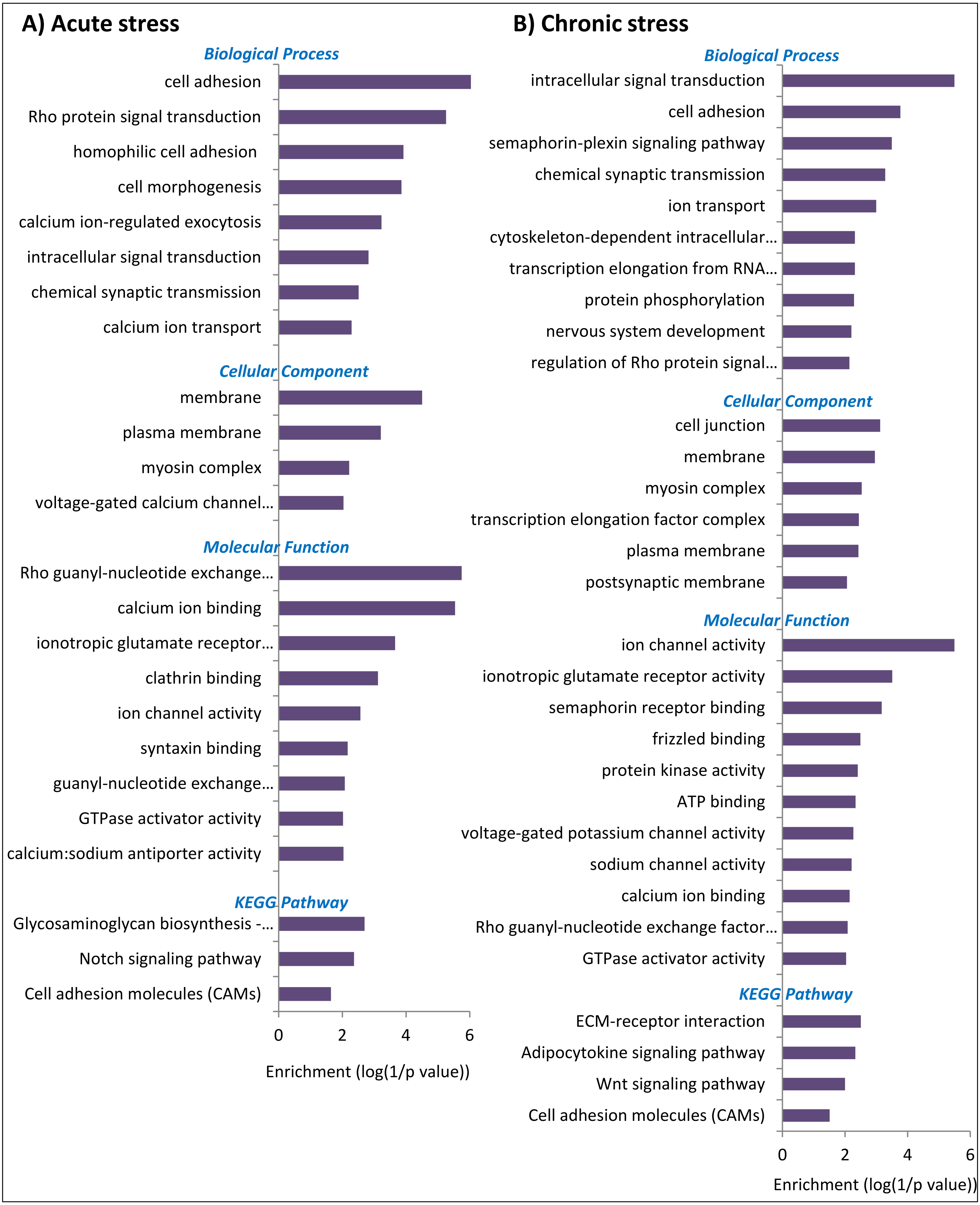

Figure S6. Gene Ontology Terms and KEGG pathways significantly over-represented (q<0.05) amongst genes that included or neighboured (2 kb upstream of TSS – 2kb downstream of TTS) differentially methylated CpGs for the acute and chronic stress groups.

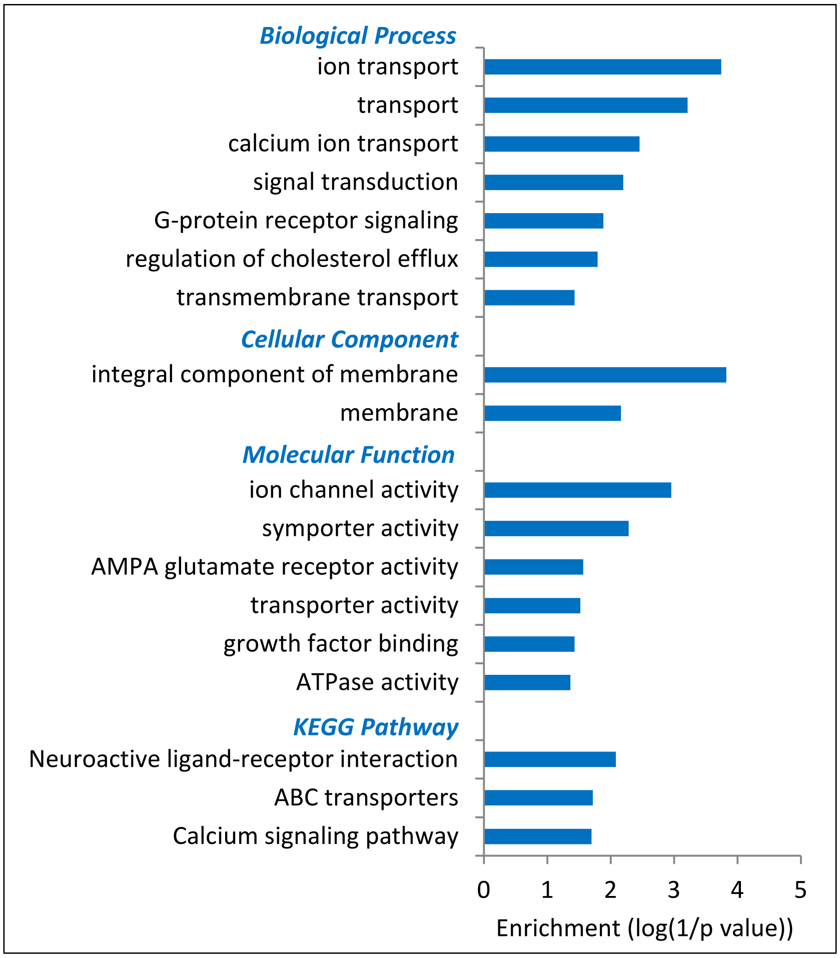

Figure S7. Gene Ontology Terms and KEGG pathways significantly over-represented ($q<0.05$) amongst genes for which a notable effect of early life stress was identified on both DNA methylation (p.promoters or gene bodies) and gene expression (>5% methylation difference and >2 FC expression).

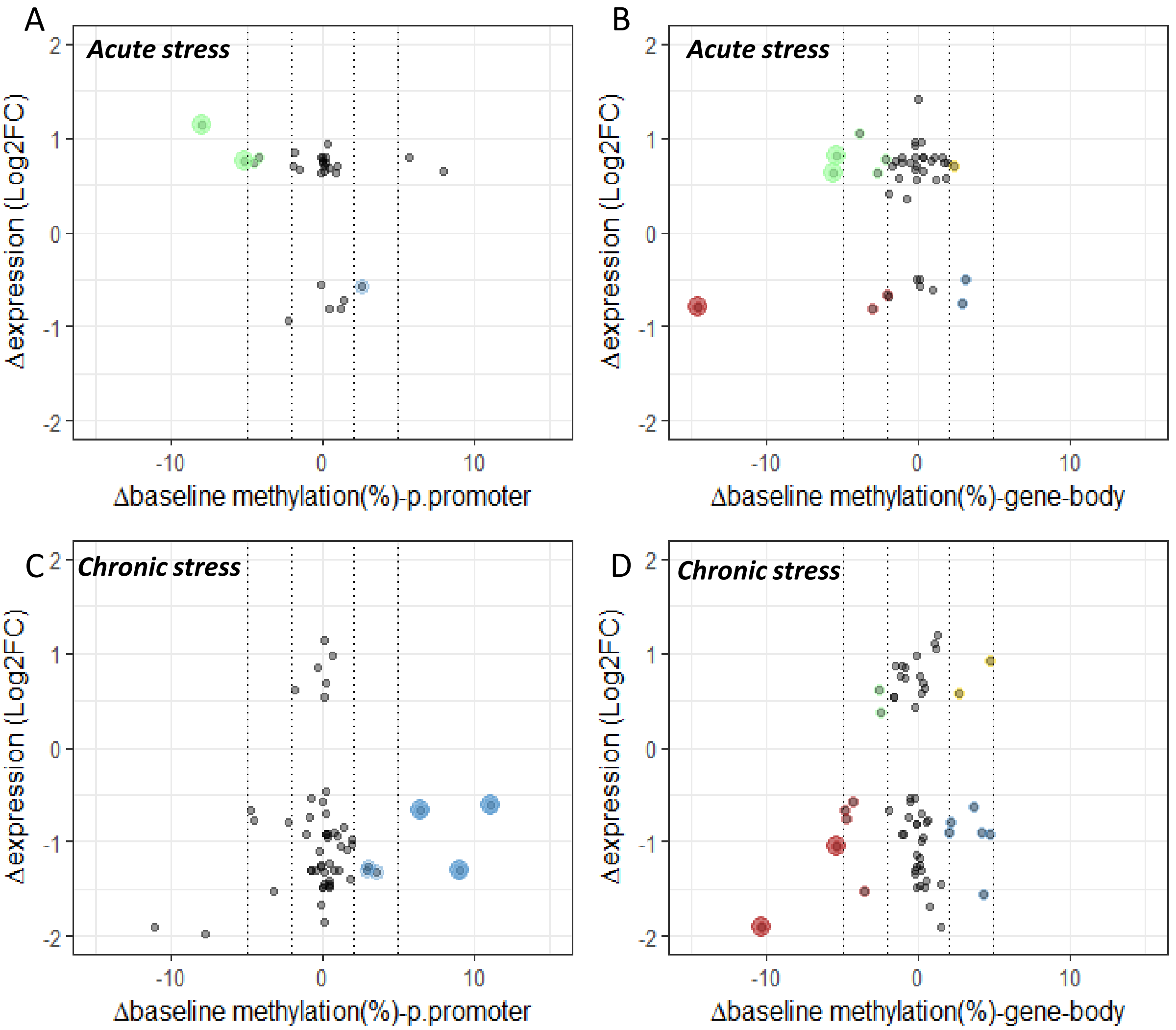

Figure S8. Integration of transcription and methylation data for genes for which a significant interaction between stress and transcriptional response to LPS was identified. The difference in LPS-responsive expression relative to the control group (Log2FC) is plotted against the difference in baseline methylation in the (A;C) putative promoter region and (B;D) gene-body for each stress group.
